# Association of herpesviruses and stroke: Systematic review and meta-analysis

**DOI:** 10.1371/journal.pone.0206163

**Published:** 2018-11-21

**Authors:** Harriet J. Forbes, Elizabeth Williamson, Laura Benjamin, Judith Breuer, Martin M. Brown, Sinéad M. Langan, Caroline Minassian, Liam Smeeth, Sara L. Thomas, Charlotte Warren-Gash

**Affiliations:** 1 Faculty of Epidemiology & Population Health, LSHTM, London, United Kingdom; 2 Institute of Infection and Global Health, University of Liverpool, Liverpool, United Kingdom; 3 Stroke Research Centre, Department of Brain Repair and Rehabilitation, UCL Institute of Neurology, UCL, London, United Kingdom; 4 UCL Division of Infection & Immunity, UCL, London, United Kingdom; University of St Andrews, UNITED KINGDOM

## Abstract

**Background:**

Herpesviruses induce a range of inflammatory effects potentially contributing to an increased risk of stroke.

**Objectives:**

To investigate whether patients with infection, or reactivation of, human herpesviruses are at increased stroke risk, compared to those without human herpesviruses.

**Data sources:**

Six medical databases and grey literature sources from inception to January 2017.

**Study eligibility criteria:**

Studies where the exposure was any human herpesvirus and the outcome was stroke. We included randomised controlled trials, cohort, case-control, case-crossover and self-controlled case series designs.

**Methods:**

Meta-analyses when sufficiently homogeneous studies were available. Quality of evidence across studies was assessed.

**Results:**

We identified 5012 publications; 41 met the eligibility criteria. Across cohort and self-controlled case series studies, there was moderate quality evidence that varicella infection in children was associated with a short-term increased stroke risk. Zoster was associated with a 1.5-fold increased stroke risk four weeks following onset (summary estimate: 1.55, 95%CI 1.46–1.65), which resolved after one year. Subgroup analyses suggested post-zoster stroke risk was greater among ophthalmic zoster patients, younger individuals and those not prescribed antivirals. Recent infection/reactivation of cytomegalovirus and herpes simplex viruses, but not past infection, was associated with increased stroke risk; however the evidence across studies was mainly derived from small, very low quality case-control studies.

**Conclusions:**

Our review shows an increased stroke risk following zoster and suggests that recent infection or reactivation of other herpesviruses increases stroke risk, although better evidence is needed. Herpesviruses are common and potentially preventable; these findings may have implications for reducing stroke burden.

## Introduction

Globally, stroke is the second most frequent cause of death.[1] There is a growing literature indicating that infections, particularly acute respiratory and urinary infections, may play a role in triggering vascular events.[2] Herpesviruses are a family of common viruses persisting latently after primary infection and reactivating periodically. The viruses induce a range of inflammatory effects,[2] potentially contributing to thrombogenesis, atherosclerosis, vasculopathy and platelet activation and thus an increased risk of stroke.

Six previous reviews support an association between herpes zoster (caused by the reactivation of varicella zoster virus (VZV)) and stroke.[3–8] One reported a risk ratio of 1.36 (95%CI 1.10–1.67) for the association between zoster and stroke pooled across six cohort studies,[4] whilst the other reviews found around 2-fold increased risk shortly after zoster, which decreased over the following year.[3, 5–7] Cytomegalovirus (CMV) is also hypothesised to modulate stroke risk, especially among immunocompromised populations[9] and a recent systematic review concluded that cytomegalovirus infection is associated with an increased risk of cardiovascular disease.[10]

Although these reviews have made a significant contribution, there are certain limitations, such as; exclusion of self-controlled case series (SCCS),[4] exclusion of studies among children,[3–8] limited subgroup analyses (only one study assessed whether antiviral therapy modified stroke risk)[7] and restricted scope by looking exclusively at clinically apparent zoster and stroke risk. Studies assessing any of the eight herpesviruses known to infect humans and utilising laboratory tests and serological analysis, as well as clinical diagnoses, could also help elucidate the role of latent, sub-clinical or clinical infection and stroke risk.

The primary objective of the systematic review was therefore to investigate whether patients with infection, or reactivation of, human herpesviruses are at increased risk of stroke,

## Methods

The protocol was published[11] according to the Preferred Reporting Items for Systematic Reviews and Meta Analyses Protocols guidelines (PROSPERO registration number:CRD42017054502).

### Study designs and characteristics

Eligible study designs included cohort, case-control, case-cohort, case-crossover and SCCS designs. Randomised controlled trials investigating prevention or treatment of herpesvirus infection or reactivation (using vaccines or antiviral agents) were also eligible. We excluded cross-sectional studies, ecological studies, case-series, case-reports and reviews. Studies were required to report an effect estimate or the data that allow its calculation. We placed no restrictions on time period, publication status, language, geographical setting or healthcare setting.

### Participants

Eligible studies included human participants. No restrictions were placed participants’ on age or immunosuppression status.

### Exposure

The exposures of interest were infection with, or reactivation of, the eight human herpesviruses: specifically, herpes simplex virus types 1 and 2 (HSV-1 and HSV-2), VZV, Epstein-Barr virus (EBV), CMV, herpesvirus 6, 7, and 8. The exposure definition could be self-reported or a confirmed diagnosis, either through clinical or laboratory criteria. Vaccination against herpesviruses (e.g. Zostavax vaccine) and treatment for herpesviruses (e.g. antivirals) were also considered as effect modifiers, to investigate whether preventing or treating human herpesviruses attenuated stroke risk.

### Comparators

Eligible studies were required to include a comparison group of people (or person time for SCCSs or case-crossover) without the herpesvirus exposure of interest.

### Outcomes

Studies were included if stroke (first ever or subsequent) was an outcome, clinically diagnosed or self-reported. Those studies meeting the inclusion criteria were additionally assessed for secondary outcomes: TIA[12] and subtypes of stroke (ischaemic versus haemorrhagic).

### Information sources

We searched for eligible articles in six databases, originally from dates of inception to January 2017, and then again in July 2018 limited to the years 2017 and 2018. The databases included Cochrane Central Register of Controlled Trials, Embase, Global Health, Medline, Scopus and Web of Science. We additionally searched the clinical trials registers (ClinicalTrials.gov) and grey literature sources, including the New York Academy of Medicine Grey Literature Report (www.greylit.org) and the Electronic Theses Online Service through the British Library (http://ethos.bl.uk).

### Search strategy

We searched medical subject heading terms and free text (in the title and abstract) for the concepts ‘human herpesviruses’ and ‘stroke’ (combined with the Boolean logic operator AND). Search terms were developed for the database Medline, reviewed by all collaborators and subsequently transcribed into search terms for the remaining databases (supplementary information S1 Appendix for search terms). Reference lists of eligible articles and relevant reviews were scanned for additional papers.

### Study selection

Eligibility assessment was performed independently in a blinded standardized manner by two reviewers (CWG and HF); all retrieved titles and abstracts were screened.

### Data collection process

Data were extracted using a pre-defined standardised template. Extraction criteria were based on the PICOS[13] (Population, Intervention, Comparator, Outcomes and Study design) framework. As this is an aetiological study, “exposure” replaced “intervention” and “study characteristics” broadened to “study design” (S3 Appendix for all items extracted). We also recorded: the most fully adjusted effect estimates (odds ratios, hazard ratios, incidence rate ratios, risk ratios) for the association between the exposure and stroke; confounders adjusted for; and results of additional analyses relevant to our non-primary objectives. If there were no events in one arm of the study, a continuity correction was applied (adding 0.5 to each cell[13]).

### Risk of bias in individual studies

Two authors independently assessed risk of bias in three studies and HF completed the remaining studies. In keeping with the Cochrane Collaborations approach,[14–16] a pre-specified set of domains were considered, including bias due to: 1) confounding; 2) selection of participants; 3) differential and non-differential misclassification of exposure and outcome; 4) missing data; and 5) reverse causation. For each domain, *a-priori* criteria were set-out to assign ‘high’, ‘low’, ‘moderate’, or ‘unclear’ risk. A summary risk of bias table was produced; when a domain had more than one item the highest risk of bias judgment was used (unless the only item at high-risk was non-differential misclassification, which would bias results toward the null).

### Synthesis of results

We synthesised the results into a narrative, grouping studies by herpesvirus exposure and study design; subgroup analyses were also described. We classified exposures as past infection or recent infection/reactivation. IgM and IgA antibodies, and DNA, are present in the blood for a limited period following herpesviruses exposure, therefore their presence suggests recent infection or reactivation (though IgM has poor sensitivity for detecting acute infections and poor specificity in immunosuppressed).[17] Conversely, IgG antibodies, although also raised during an acute infection or reactivation, remain during latent infection, therefore were classified as a past infection.[17] We also presented results from studies of high versus low IgG titre, as high IgG titre may reflect recent reactivation.

When at least two studies assessed the same herpesvirus as a stroke risk factor, meta-analysis was considered. For pooling, we required studies to have identical study designs, the same measurement for the herpesvirus (e.g. IgG seropositivity) and identify the outcome within a similar time-frame. We pooled effect sizes (referred to as “summary estimates”) irrespective of the type of effect estimate, due to stroke being rare. Random effects meta-analysis was used throughout, to ensure a consistent approach to all analyses was employed; the I^2^ statistic indicated moderate heterogeneity (I^2^>25%) for many subgroups. We investigated sources of heterogeneity (where there were at least three studies in the meta-analysis) by removing studies at high-risk of bias.

### Quality of the evidence

The Grading of Recommendations, Assessment, Development and Evaluation (GRADE)[18] approach was used to summarise the quality of cumulative evidence for each herpesvirus on stroke. Evidence was categorised as ‘high’, ‘moderate’, ‘low’ or ‘very low’ quality, with observational studies starting as ‘low’; five reasons to rate down and three reasons to rate up the quality of evidence, were then considered.[19] Full criteria for grading is in S4 Appendix. We assessed publication bias when there were at least 10 studies by creating a funnel plot: effect estimates for the exposure on stroke risk were plotted against standard errors of the log odds, and symmetry was assessed visually and using Begg’s test for small-study effects.[20]

### Ethics

As this is a systematic review, ethical approval is not required.

## Results

**Fig 1:**
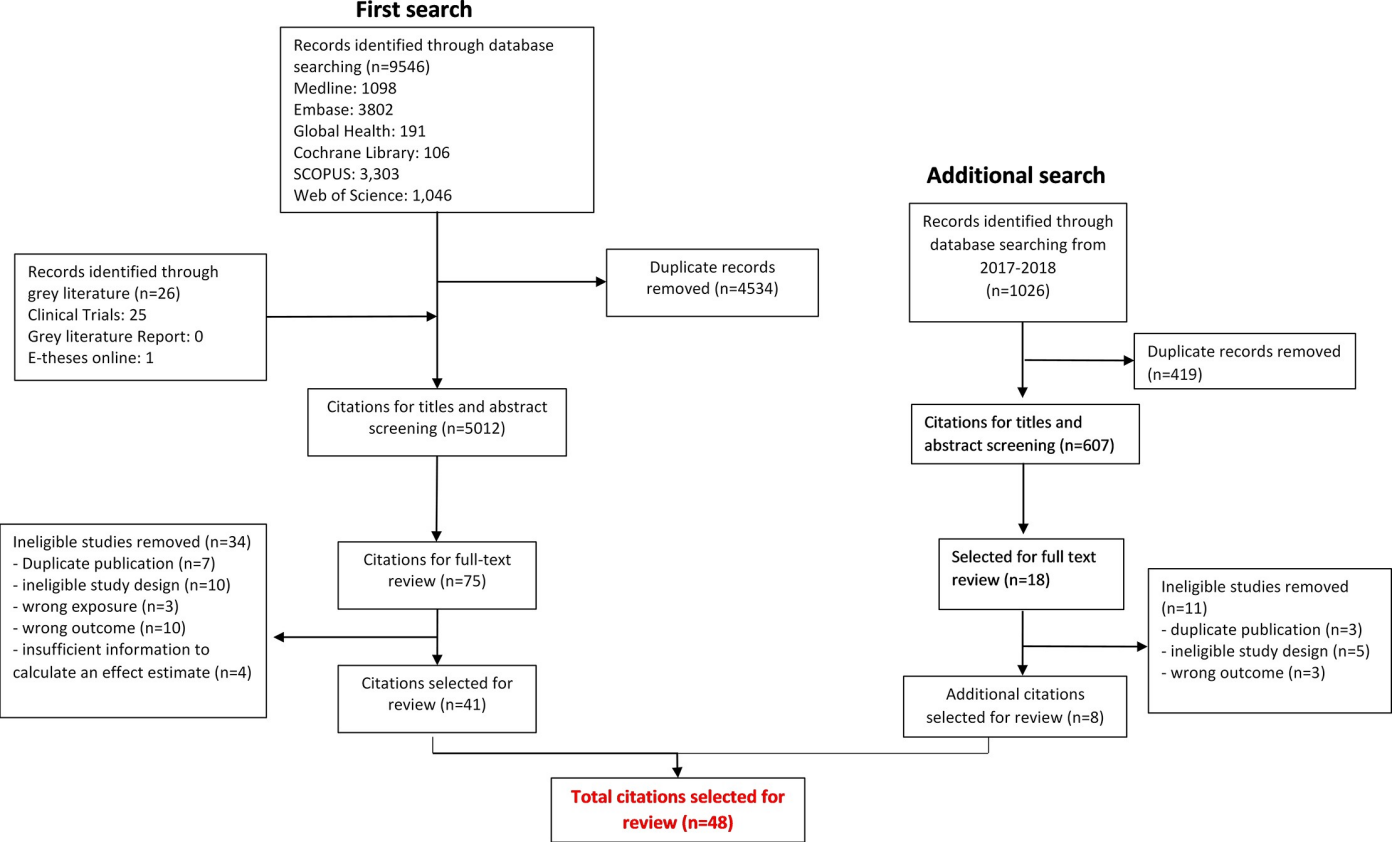
Flow diagram of study selection.

In our initial search 5012 titles and abstracts were screened and 41 observational studies were selected for review (Fig 1). Our updated search retrieved 607 studies, of which seven were selected for review, making a total of 48 studies for the final review.

**Fig 2:**
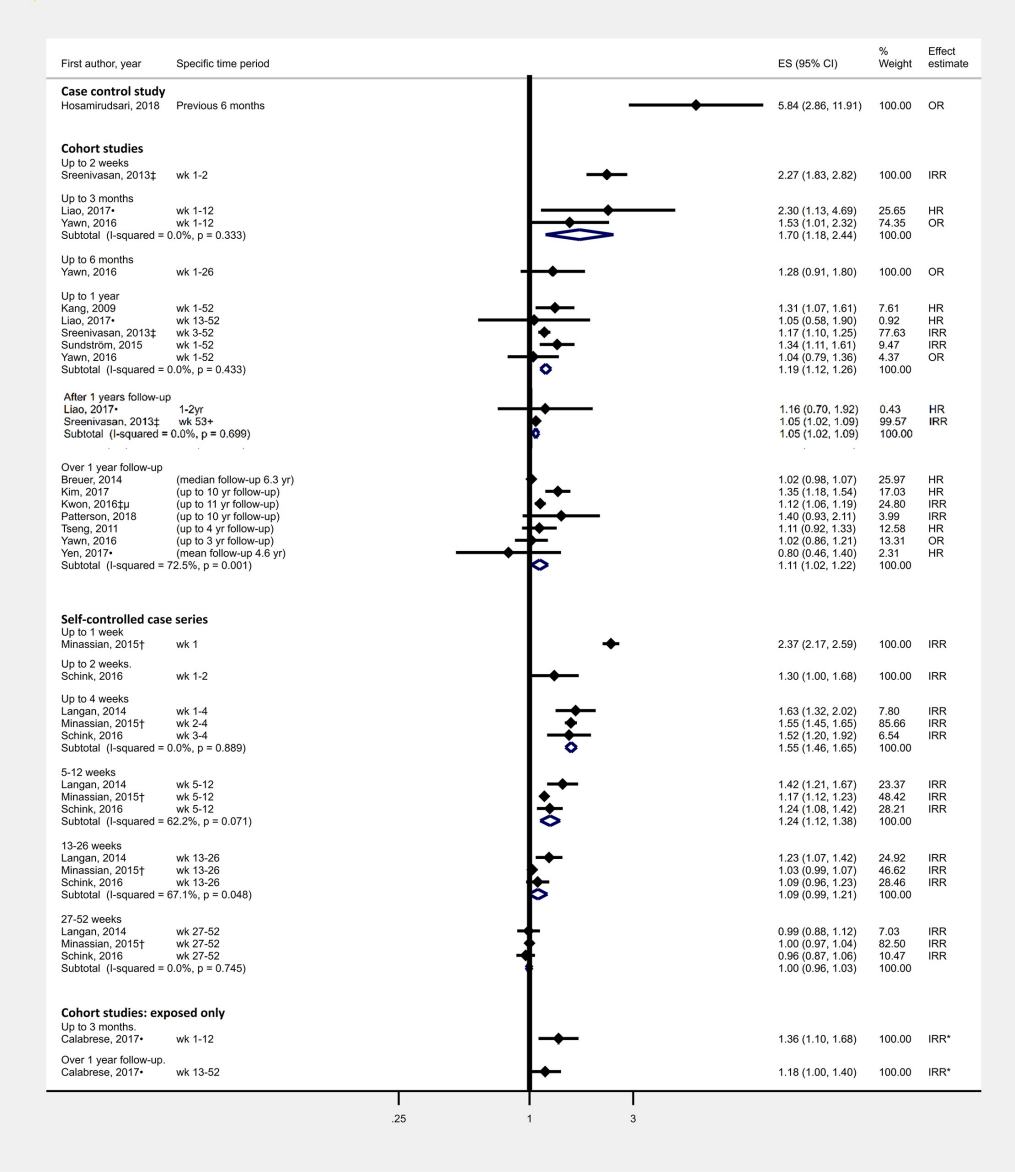
Effect of clinically diagnosed herpes zoster on stroke risk by study design and length of follow-up. †Outcome was ischaemic stroke ‡Outcome was stroke/TIA μ: among patients 50–60 years of age. •Study population was immunosuppressed *Comparator group was person time 366-730days after HZ.

**Fig 3:**
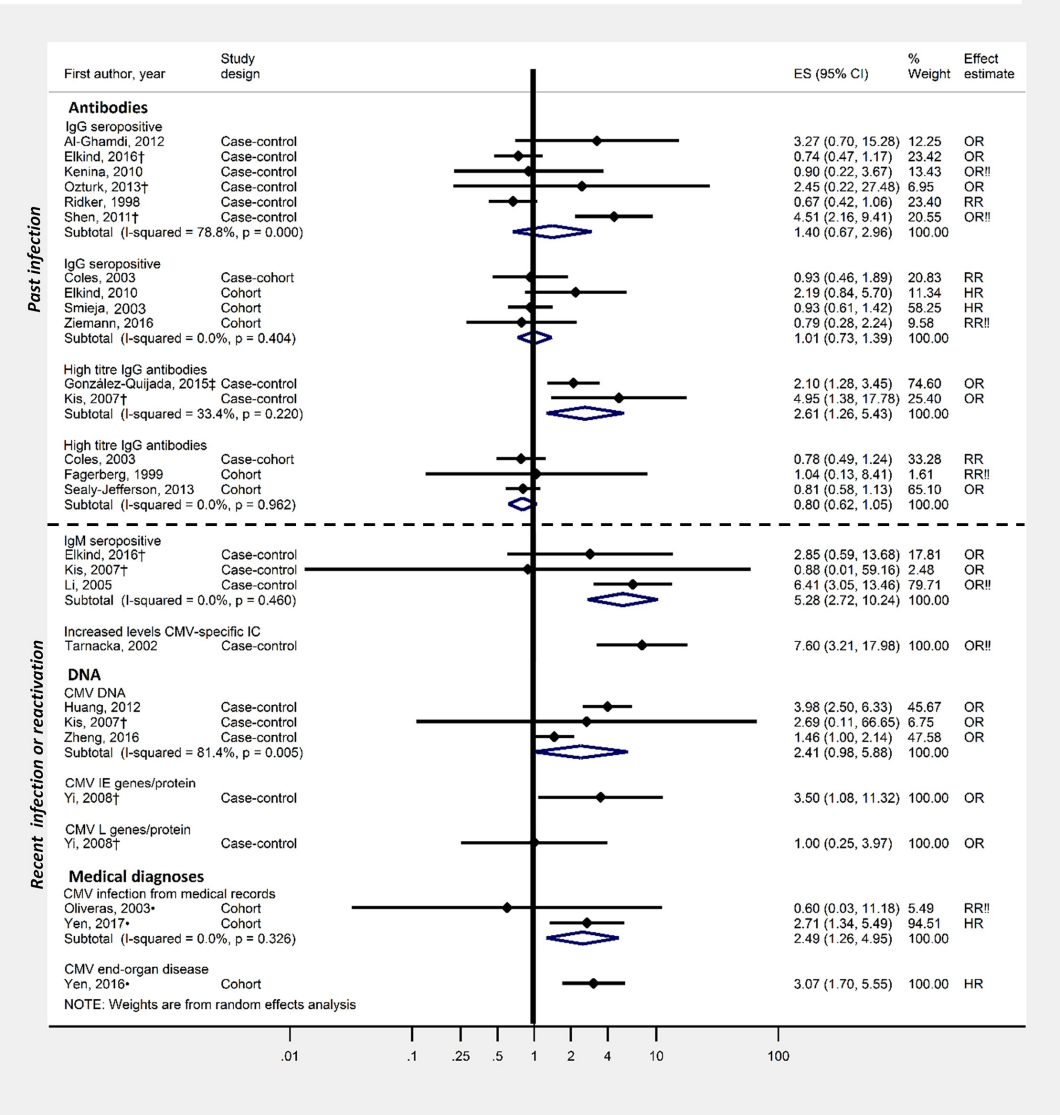
Effect of CMV (serological evidence of infection or clinical reactivation) on stroke risk. †Outcome was ischaemic stroke ‡Outcome was stroke/TIA •Study population was immunosuppressed. ‼No age adjustment/matching for age.

**Fig 4:**
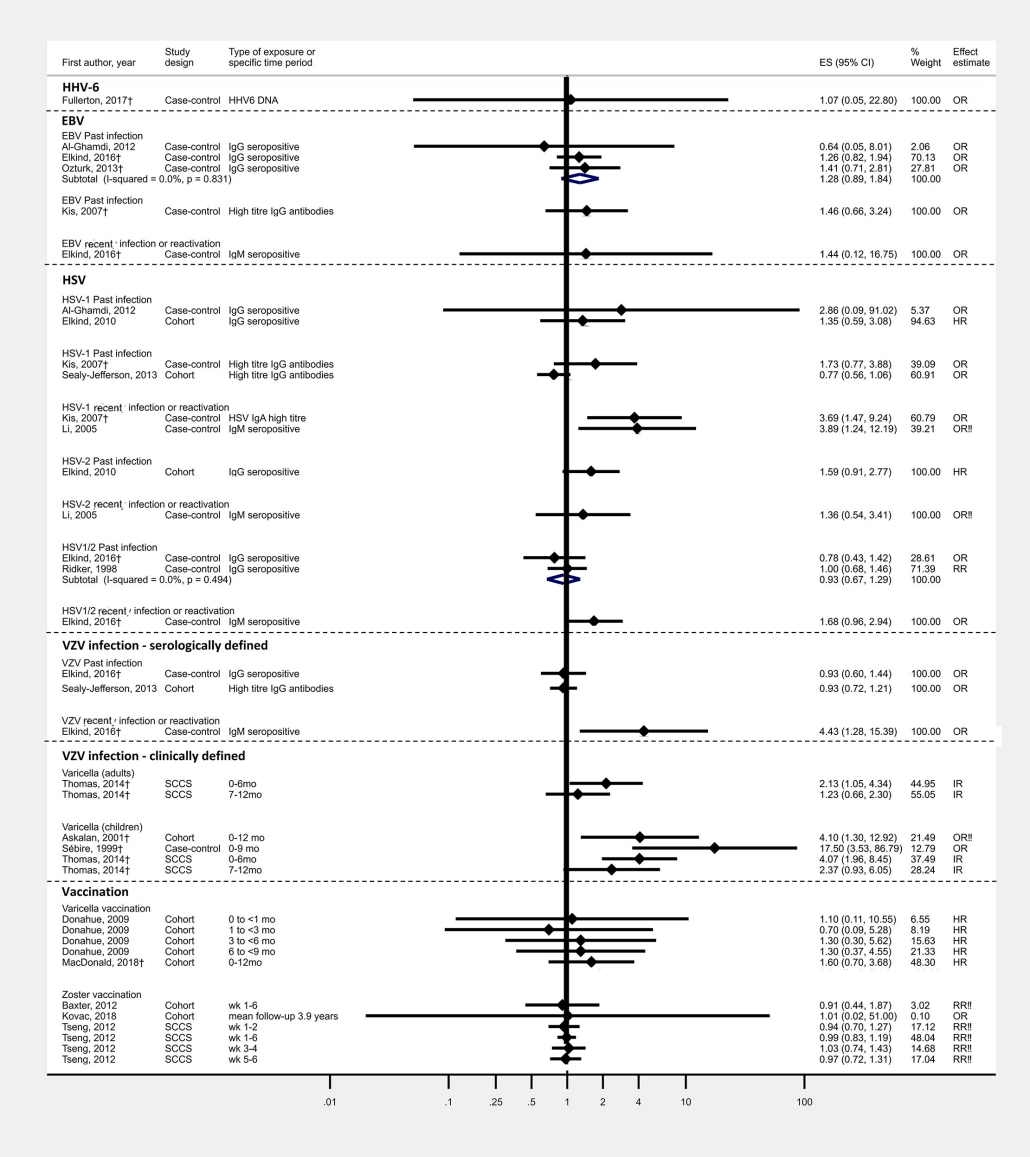
Effect of EBV, HSV, VZV infection, clinically diagnosed varicella and VZV vaccination on stroke risk. †Outcome was ischaemic stroke ‡Outcome was stroke/TIA ‼No age adjustment/matching for age.

Study methods and results are summarised in Tables 1 and 2 respectively, risk of bias for individual studies in Table 3 (S5 Appendix for detailed justification) and GRADE assessment in Table 4. Results and meta-analyses are displayed in Figs 2–4.

**Table 1:** Study characteristics.

Author, yr	Design	Study period	Setting	Study population at recruitment	Exposure definition and ascertainment	Comparator definition and ascertainment	Outcome type	Outcome definition and ascertainment
**VZV reactivation—Herpes zoster**								
Breuer, 2014[22]	Cohort	2002–2010	UK, primary care records from THIN	Adults (≥18 yr) with HZ and age (± 2 yr), sex and GP practice matched (2:1) patients with no HZ.	Non-recurrent HZ: Read codes	Patients without an HZ Read code	Primary: stroke or TIA Secondary: ischaemic, haemorrhagic or unspecified stroke	First ever incident stroke or TIA: Read codes
Calabrese, 2017[23]	Cohort	2006–2013	United States, Medicare claims data	Adults ≥65 yr with HZ, ≥12mo follow-up at entry, inflammatory disease (ankylosing spondylitis/ IBD/ psoriasis/ psoriatic arthritis/RA), no prior stroke or antiviral therapy	Inpatient/outpatient HZ: ICD-9 diagnosis code AND no same day code for zoster vaccine	Time after HZ divided into 3 periods: 0–90 days; 91–365 days; 366–730 days (reference group).	Primary: Any stroke Secondary: ischaemic stroke	Hospitalised stroke: ICD-9 diagnosis code in any position on hospital claim.
Hosamirudsari, 2018 [21]	Case-control	2015–2017	Iran, individuals attending a single hospital	Adults (aged 30–90 years) admitted for stroke, and controls were stroke-free individuals	Self-reported HZ infection in the last 6 months, collected by a team of healthcare specialists.	No self-report of HZ infection	Stroke	Stroke diagnosed by neurologist and confirmed by brain imaging
Kang, 2009[24]	Cohort	1997–2001	Taiwan, National Health Research Institute claims database	Patients (≥18 yr) with HZ and no history of stroke, matched to 3 patients (age, sex) with no history of HZ or stroke before 2001.	Treatment for HZ in ambulatory care: ICD-9 codes.	Patients without a history of HZ	Primary: stroke (any) Secondary: ischaemic, haemorrhagic	ICD-9 codes
Kim, 2017[32]	Cohort	2002–2013	Korea, sample of national health insurance database	Patients (age unknown) with HZ propensity score matched to those without HZ.	HZ diagnosis: ICD-10 codes	Patients without HZ	Stroke	Newly diagnosed stroke: ICD-10 codes
Kwon, 2016[25]	Cohort	2003–2013	Korea, 1 million sample of national health insurance database	All patients (>18 yr) in database: those with HZ or stroke during 1st yr of observation period excluded.	First HZ in the observation period: ascertained from ICD-10 codes.	Patients without a history of HZ.	Stroke/TIA	First ever stroke or TIA: ICD-10 codes
Langan, 2014[37]	SCCS	1987– 2012	UK, CPRD; routinely collected database of primary and secondary care records.	Adults (≥18 yr) with 1st ever HZ and stroke. Exclusions: incident TIA, subarachnoid haemorrhage, encephalitis in 12 mo after stroke.	1st ever HZ: Read and ICD-10 codes. Exposed period: day after HZ to 12 mo (wk 1–4, 5–12, 13–26, and 27–52).	All observation time around exposed period, with the exception of the day of HZ and 4-wk pre-HZ	Primary: Arterial stroke Secondary: cerebral infarction, haemorrhagic or unspecified stroke	First ever stroke: Read codes in CPRD and ICD-10 codes in linked hospital data.
Liao, 2017[33]	Cohort	2000–2011	Taiwan, National Health Research Institute claims database	Adults (≥18 yr) with rheumatoid arthritis. Those with HZ matched (on age, sex, disease duration) to those without HZ. Excluded those with HZ or stroke prior to entry	HZ diagnosis after study entry: ICD-9 codes.	Patients without HZ	Stroke	ICD-9 codes
Lin, 2010[26]	Cohort	2003–2005	Taiwan, National Health Research Institute claims database	Immunocompetent adults (≥ 18 yr) with HZO, matched (age, gender) to 3 without HZO. Excluded those with stroke prior to entry.	Patients seeking ambulatory care for HZO (patients with HZO in the previous yr excluded: ICD-9 code (053.2)	Patients without HZ. First ambulatory care visit in 2004 was assigned their index date.	Stroke	Not specified: most likely from ICD-9 codes
Minassian, 2015[35]	SCCS	2006–2011	United States, Medicare claims data	Patients (≥65 yr) with HZ and stroke / TIA. Excluded if had HZ or vascular events pre-entry or subarachnoid haemorrhage ever or encephalitis in 12 mo post-stroke	HZ episode; ICD-9 code with antiviral 7 days before or after HZ. Exposed period: 12-mo after HZ (wk 1, wk 2–4, 5–12, 13–26, and 27–52).	All other observation time made up the baseline (unexposed) period, except the day of and the 4 wk before HZ diagnosis.	Primary: ischaemic stroke Secondary: haemorrhagic stroke	Stroke: ICD-9 codes in outpatient and inpatient (primary diagnostic field) records
Patterson, 2018[34]	Cohort	2007–2014	United States, Medicare and MarketScan data	Adults (> = 18) at HZ diagnosis, propensity matched to HZ-free controls.	HZ diagnosis	Patients without HZ	Stroke and TIA	Not specified: most likely from ICD-9 codes
Schink, 2016[36]	SCCS	2004–2011	Germany,health claims data from 4 insurance providers, hospitalisations and outpatients data	Patients (any age) with HZ and stroke, ≥12 mo follow-up, no history of stroke or HZ in 12 mo pre-cohort entry.	1st or recurrent HZ: ICD-10 code or antiviral with HZ outpatient-diagnosis. Exposed period: up to 12 mo from HZ (0–2 wk, 3–4 wk, 2–3 mo, 4–6 mo, 7–12 mo)	All follow-up time outside exposed period.	Primary: First stroke Secondary: ischaemic, haemorrhagic, stroke unspecified or TIA.	Hospitalised stroke: ICD-10 codes for main discharge diagnosis in hospitalisation data. Admission date taken as onset date
Sreenivasan, 2013[27]	Cohort	1995–2008	Denmark, routinely collected civil registration data and health registers.	All Danish adults (≥18 yr) alive during study period. Persons with outcome before start of follow-up were excluded.	HZ treated with antivirals; acyclovir prescription (800 mg in packages of 35 tablets)	Cohort members with no prior history of acyclovir, valacyclovir or famciclovir prescriptions.	Stroke and TIA (as a composite outcome)	ICD 8 and 10 codes, from National Patient Registry; a database of all hospitalisations, outpatient visits and emergency department visits.
Sundström, 2015[28]	Cohort	2008–2010	Sweden, routinely collected healthcare data from one county.	All incident cases of HZ occurring during the study period and the general population in the country. No age restrictions.	HZ from ICD-10 codes, with no diagnosis of HZ in the previous yr.	General population in the country (no further information given).	Stroke	ICD-10 diagnosis within 1 yr of HZ diagnosis.
Tseng, 2011[65]	Cohort	2007–2010	United States, Kaiser Permanente Southern California health care	HZ cases (≥50 yr) without history of stroke 1 yr pre-HZ, matched (age, date of HZ, setting of medical care) to patients without HZ	HZ cases who had received treatment for HZ during the study period	Patients without HZ	Stroke	Incident stroke, identified from hospitalisation records with a primary diagnosis as stroke.
Yawn, 2016[31]	Cohort	1986–2011	United States, medical records from Olmsted County.	All adults (≥50 yr) with HZ, matched (sex, age (+/- 1 yr)) to patients without HZ. Patients with history of stroke excluded.	1st/recurrent HZ; ICD-9 code and HZ clinical symptoms in medical records	Patients with no HZ diagnoses in five yr prior to cohort entry.	Stroke	Diagnostic codes from hospital admissions or death records, <30 days before cohort entry, or until cohort exit.
**CMV infection**								
Coles, 2003[38]	Case-cohort	1995–1998	Australia, Busselton Health Survey, and linked hospital and death data	Adults (40–89 yr) developing stroke and random sample of non-stroke adults, without CVD at baseline, with serum samples from 1981.	IgG antibodies: avidity assay (AxSym) High level sample = ≥250.	Participants without IgG antibodies to CMV	Stroke	First stroke; from ICD-10 codes and defined as either admission to hospital with any diagnosis of stroke or death from stroke.
Fagerberg, 1999[39]	Cohort	1987–1995	Sweden, men from intervention study with hypertension and ≥1 other CVD risk factor.	Men (50 to 72 yr). Of 508 recruited into intervention study, 164 (32%) randomly selected to participate in sub-studies.	High IgG antibodies: MEIA on serological samples taken at entry and/or 3.5 yr later. High titre undefined	Participants with low titres against CMV.	Non-fatal stroke	Independently coded by 2 physicians using hospital records, autopsy records, and death certificates.
González-Quijada, 2015[40]	Case-control	2011–2013	Spain, random sample of elderly patients from a single hospital	Cases (stroke patients) and controls (non-stroke patients) aged ≥65 yr (unmatched).	High IgG antibodies: ELISA. Defined as top quartile of serological values. Date samples taken unknown.	CMV seropositive participants without high-titre IgG antibodies.	Ischaemic stroke or TIA	Prevalent or incident ischaemic stroke and/or TIA: determined by imaging data or neurology / internal medicine specialists.
Huang, 2012[41]	Case-control	1997–2000	China, Stroke Hypertension Investigation in Genetics case-control study	Stroke patients matched to controls without stroke (sex, age ±3 yr, geographic location, blood pressure category. Age unknown.	IgG, IgM antibodies: ELISA DNA: PCR on plasma samples taken after stroke diagnosis (date unknown).	Participants without any CMV infection	Primary: Any stroke Secondary: ischaemic and haemorrhagic.	Stroke patients discharged from hospital with stroke in past 5 yr. Diagnosed by computer tomography or magnetic resonance imaging
Kenina, 2010[42]	Case-control	Unclear	Latvia, single hospital. Data collected through clinical evaluation and questionnaires.	Stroke patients and controls aged ≥42 yr.	IgG antibodies:plasma and sera using ELISA	Participants without any CMV infection	Primary: ischaemic Secondary: Atherotrombotic, Cardioembolic or Undetermined	Stroke patients hospitalised in the Clinic of Neurology
Oliveras, 2003[43]	Cohort	1979–2000	Spain, single hospital. Data collected retrospectively.	Patients who received renal transplants between 1979–2000.	CMV infection from medical records: no further information provided	Participants without any CMV infection	Primary: Any stroke Secondary: ischaemic and haemorrhagic.	Diagnosis based on clinical symptoms and brain CT scan or MRI.
Shen, 2011[44]	Case-control	2009	China, inpatients and outpatients from neurology department of single hospital	Cases (aged ≤75 years) with cerebral infarction and controls with a noraml carotid ultrascan scan and cerebral CT/MRI scan.	IgM antibodies: ELISA techniques from serum samples.	Participants without any CMV infection	Ischaemic stroke	Diagnosis based on the 1995 National Cerebrovascular Disease Meeting standard for cerebral infarction, combined with a CT/MRI scan.
Smieja, 2003[45]	Cohort	1993–1995 (recruitment)	Canada, multicentre RCT among patients with history of CVD	Patients ≥55 yr with blood samples (N = 3168/9541). Excluded those with;MI/ stroke 4 wk before study.	IgG antibodies: quantitative CMV IgG assay. Samples taken at baseline.	Participants with no evidence of CMV infection	Stroke (secondary outcome)	Stroke was defined as a neurologic deficit lasting more than 24 hours
Tarnacka, 2002[46]	Case-control	1998–1999	Poland, patients with stroke consecutively admitted to neurology department.	Cases were patients with stroke. Two control groups; “old” and “young,” no clinical signs of infection/ other systemic diseases/ ischaemic stroke. All had increased values of serum IC concentrations	Elevated levels of IC containing anti-CMV antibodies; ELISA. Blood samples taken <24 hrs and 7 to 30 days after stroke onset.	Elevated levels of IC not containing anti-CMV antibodies	Ischaemic stroke	Stroke within 24 hrs after onset. CT imaging, sonography, echocardiography, and laboratory tests confirmed the diagnosis, established from history and examination
Yi, 2008[47]	Case-control	Unclear	China, no further information	Cases (≥50 yrs) died of stroke, matched (age, sex) to controls with no cerebro-vascular disease, CMV-associated disease, immune suppression, or IgG for CMV.	DNA: immediate early (IE) and late (L) antigen in the intracranial arteries by PCR	Participants without CMV DNA	Ischaemic stroke	Patients died of ischaemic stroke.
Zheng, 2016[48]	Case-control	2004–2014	China, cohort study within a rural population with hypertension	Random sample of stroke cases (≥35 yr), matched (age [1 yr], sex, duration follow-up, hypertension stage) to controls without stroke. Patients with stroke and CAD at baseline excluded.	DNA: PCR on blood samples taken at recruitment to original cohort study (prior to stroke).	Participants without CMV DNA. A negative result meant no DNA was detected.	Stroke	First ever stroke during follow-up: evidence from imaging data extracted from patients medical records, and independently reviewed by the end-point assessment committee.
Ziemann, 2016[49]	Cohort	2008	Germany, trial in patients undergoing cardiac surgery, from single hospital	Patients (≥18 yr) due for cardiac surgery: those with planned off-pump surgery excluded. 195/1178 (16.6%) patients excluded due to inadequate blood samples.	IgG antibodies: CMIA DNA: PCR, arterial blood samples taken just before induction of anaesthesia.	CMV seronegative participants	Stroke	Derived from the prospectively sampled cardiac surgery database. Defined as Stroke with Rankin grade>1.
**CMV reactivation**								
Yen, 2016[9]	Cohort	1998–2012	Taiwan, National Health Insurance Research Database.	Adults (≥15 yr) newly diagnosed with HIV, with no history of stroke or CMV infection.	CMV end-organ disease: ICD-9 code (0.78.5 Cytomegaloviral disease) and prescription for an anti-CMV drug	Participants without CMV end-organ disease	Primary: Any stroke Secondary: ischaemic and haemorrhagic.	Hospitalisation or stroke from ICD-9 codes
**HHV6 infection**								
Fullerton, 2017[58]	Case-control	2009–2014	United States,	Children (aged 28 d to 18 yrs) with stroke and stroke-free trauma controls, frequency matched on age.	HHV6 DNA: MassTag PCR	Participants without HHV6 DNA	Ischaemic stroke	Acute diagnosis of ischemic stroke
**VZV infection, serologically defined**								
Asiki, 2015[59]	Case-control	Unclear	Uganda, data from population based cohort study in rural setting	Adults stroke patients matched on sex and age to ≥4 controls without stroke. All participants had stored serum samples.	IgG, IgM antibodies: quantitative indirect chemiluminescent immunoassays at/prior to stroke	IgG and IgM optical densities compared in cases versus controls	Stroke	Prevalent cases from clinical symptoms and deaths due to stroke by verbal autopsy.
**VZV infection, clinically defined (varicella)**								
Askalan, 2001[60]	Cohort	1992–1999	Canada, two hospitals.	Consecutive young children aged 6 mo to 10 yr with acute (or unders 3–6 mo follow-up for) stroke during the study period.	Varicella 12 mo prior to stroke: parental interview	Patients without varicella	Recurrent cerebral ischaemic events	TIAs and cerebral infarctions before or after the index AIS: parental interviews, radiographic films, health-record review
Sébire, 1999[61]	Case-control	1985–1996	France, referrals to single hospital for stroke treatment	Children with stroke matched to 4 healthy children (gender, age (±4 mo), site of residence)	Varicella in the 9 mo prior to stroke; from an obligatory French health record	Participants without varicella	Ischaemic stroke	First idiopathic arterial ischaemic stroke: angiograms and long-term clinical and angiographic follow-up
Thomas, 2014[62]	SCCS	1990–2011	UK, primary and secondary care records from 4 routinely collected databases	Patients (any age) with first ever stroke/TIA and chickenpox during study period.	Varicella: from Read codes. Exposed period: day after varicella and up to 1 yr after	"Unexposed" time: all follow-up time when individual not "exposed".	Ischaemic stroke	First stroke within study period; ascertained from Read codes.
**Vaccination against herpesviruses**								
Baxter, 2012[64]	Cohort	2006–2007	United States, Kaiser Permanente Northern California (KPNC), health care system	People (≥60 yr) vaccinated against HZ in routine medical care, with ≥180 days continuous KPNC membership after vaccination.	HZ vaccination: in Kaiser Immunization Tracking system. Exposed period: 1–42 days after vaccination.	Unexposed period: 91–180 days post vaccination.	Stroke	Evidence of stroke (hospitalisations and emergency department visits) in 1–42 days following vaccination
Kovac, 2018[63]	RCT		Multi-country, randomised placebo-controlled trial	People(≥50 yr) randomised to placebo or HZ subunit vaccine. Excluded those with history of zoster, VZV vaccination, an immunosuppressive condition.	HZ subunit vaccination	Placebo vaccination.	Stroke	Clinical evidence of stroke (neurological deficit and change in consciousness) and either CT/MRI scan or no other sign of a disorder causing deficits
Tseng, 2012[65]	SCCS	2007–2008	United States, 8 managed-care systems taking part in Vaccine Safety Datalink Project	Patients ≥50 yr receiving HZ vaccine who experienced stroke. 12 mo continuous membership was required, prior to first event.	HZ vaccination: medical records. Risk windows: 1–14 days, 15–28 days, 29–42 days, 1–42 days from vaccination.	Same length of time after a 30-day "wash-out" period following the risk window.	Stroke	ICD-9 diagnosis codes from inpatients and emergency department records, with no code in the previous 12 mo
Donahue, 2009[66]	Cohort	1991–2004	United States, 8 managed-care systems taking part in Vaccine Safety Datalink Project	Immunocompetent children (11mo to 17yr), ≥12 mo continuous enrolment, ≥1 encounter with site. Excluded those with infantile cerebral palsy stroke, or hemiplegia/ hemiparesis at ≤11 mo of age	Varicella vaccination: recorded in Vaccine Safety DataLink database. Exposed period: 12-mo period following vaccination.	1) children without varicella vaccination; 2) exposed children: all other person time not classified as exposed.	Ischaemic stroke	Primary or secondary coded diagnoses in inpatient settings using ICD-9 codes
MacDonald, 2018[67]	Cohort	2006–2013	Canada, administrative health databases	Children receiving the varicella vaccination between 11 months and 23 months of age, and non-vaccinated children.	Varicella vaccination: recorded in medical records. Exposed period: 12-mo period following vaccination.	Children without varicella vaccination	Ischaemic stroke	ICD-10 codes recorded in hospital discharge database
**Multiple herpesviruses infections**								
Al-Ghamdi, 2012[50]	Case- control	Unclear	Saudi Arabia, a single hospital setting	Patients with atherosclerotic vascular disease, matched (age, sex) to 15 healthy controls. Age not specified.	HSV-1 and EBV IgG antibodies: ELISA kits. CMV IgG antibodies: bioelisa kit.	Participants with a negative test result for exposures	Stroke	Not reported
Elkind, 2010[52]	Cohort	1993–2001	US, community-based study to investigate epidemiology of stroke.	Adults >39 yr, with no history of stroke, residing in household with a telephone, with blood samples available.	CMV, HSV-1 and HSV-2: Enzyme-linked immunoassay used to measure IgG antibody titres against exposures.	Participants with a negative test result for exposures	Stroke	Defined using data from annual telephone follow-ups: symptoms and events consistent with stroke and classified by 2 neurologists.
Elkind, 2016[51]	Case-control	2010–2014	9 countries, Vascular Effects of Infection in Paediatric Stroke study	All children (29 days to 18 yr) presenting to an included centre and enroled ≤3 wk of stroke, with an analysable blood sample.	IgG, IgM antibodies to HSV-1/2, CMV, EBV, VZV: blood samples ≤3 wk from stroke using ELISAs. Clinical infection, previous 6 mo from parent/guardian interview.	Participants without evidence of infection.	Ischaemic stroke	Arterial ischaemic stroke: from clinical and imaging data by a trained specialist.
Kis, 2007[53]	Case-control	2003	Hungary, patients hospitalised in 2003	Cases (<65 yr) admitted <72 hr after stroke. Controls (<76 yr) admitted for pain, without ischaemic stroke. Patients with history of MI, atrial fibrillation, valvular or myocardial heart disease excluded.	CMV DNA: PCR IgG, IgA, IgM antibodies to HSV-1, CMV, EBV and HHV-6: ELISA, blood samples taken ≤1 wk from stroke.	Participants without evidence of infection.	Ischaemic stroke	First noncardiogenic ischaemic stroke: from clinical examinations and imaging techniques.
Li, 2005[54]	Case-control	2001–2002	China, department of neurology in a single hospital	Cases (age unspecified) of stroke in progression. Excluded those with embolism and reversible ischaemic neurological deficit, cerebral haemorrhage, haemorrhagic infarction, >5 yr history of severe disease. Controls were patients with non-cerebrovascular disease.	CMV, HSV-1 and HSV-2 IgM: dot immunogold labelling staining performed after stroke diagnosis (date unknown).	Patients without herpesvirus IgM in blood	Stroke in progression	Brain damage caused by an obstruction to the blood supply not preventable with convention treatment (e.g. urokinase for injection) within 72 hours from stroke onset. Confirmed with CT and/or MRI.
Ozturk, 2013[55]	Case-control	Unclear	Turkey, department of neurology in a single hospital	Cases (>40 yrs) were patients presenting with stroke <24 hours of onset matched (age) to controls without ischaemic stroke or TIA. Patients with TIA, subarachnoidal hemorrhage, cerebral venous sinus occlusion with ischemic stroke due to head trauma were excluded.	CMV, EBV IgG: blood samples. CMV tested using ELISA and EBV tested using Viral capsid antigen	Patients without herpesvirus IgG in blood	Ischaemic stroke	Sudden focal or global cerebral impairment and at least one acute lesion. Computed tomography (CT) and magnetic resonance imaging (MRI) were performed in all patients during the first 24 hours
Ridker, 1998[56]	Case-control	Unclear	US, Physicians Health Study (RCT among male doctors with no history of MI, stroke or cancer).	Cases (age unspecified) were patients developing stroke/MI, matched (age, smoking, follow-up) to controls without MI or stroke. Participants with baseline blood samples included (14916/22071 [68%]).	CMV, HSV1/2 infection: plasma assayed using ELISA for presence or absence of IgG antibodies directed against HSV and CMV.	Seropositivity was compared in cases versus controls	Thromboembolic stroke	Hospital records and autopsy reports were used to confirm each diagnosis according to prespecified criteria
Sealy-Jefferson, 2013[57]	Cohort	1998–2008	US, cohort of Mexican Americans from the Sacramento Area Latino Study on Aging, community-dwelling	Participants from the cohort (60–101 yr at baseline) without a history of stroke at baseline.	CMV, HSV-1 and VZV IgG antibodies: solid-phase ELISA. Measured at baseline and follow-up visits	Seronegative to herpesviruses of interest.	Incident stroke	Self-reported: determined at follow-up visits and semi-annual telephone calls. Fatal strokes identified from death certificates using the ICD-10 code 164.
Yen, 2017[30]	Cohort	2000–2012	Taiwan, National Health Insurance Research Database.	Patients (≥15 yr) with new HIV diagnosis. Patients who received a stroke diagnosis were excluded.	HZ and CMV disease after HIV diagnosis: ICD-9 codes from an inpatient setting or in ≥3 outpatient visits.	Participants without diagnosis codes for HZ or CMV.	Stroke	Patients hospitalised for stroke, identified through ICD-9 codes

Abbreviations: RCT = randomised controlled trial, SCCS = self-controlled case series, RR = risk (or rate) ratio, CI = confidence interval, transient ischaemic attack = TIA, HZ = herpes zoster, HZO = herpes zoster opthalmicus, ESRD = End-stage renal disease, CT = computerised tomography, MRI = magnetic resonance imaging, IBD = inflammatory bowel disease, SLE = systemic lupus erythematosus, MS = multiple sclerosis, RA = rheumatoid arthritis, HMO = health maintenance organization, GPRD = General Practice Research Database, THIN = The Health Improvement Network, CVD = cardiovascular disease, MEIA = micro-particle enzyme immunoassay technique, CMIA = chemiluminescent microparticle immunoassay, CAD = coronary artery disease, MI = myocardial infarction, ACE = angiotensin-converting–enzyme, IU = International Units, yr = year, mo = mo, wk = wk, hr = hour

*Or Odds Ratio (OR) if specified.

**Table 2:** Study results.

First author, publication yr	Design	Population size (N), follow-up time (yr)	Subjects with outcome [or exposure for case-control studies] (N, %)	Statistical analysis method used	Main reported results	Adjusted for
**VZV reactivation—herpes zoster**						
Breuer, 2014	Cohort	Exposed = 106,601 Unexposed = 213,202 Follow-up (median): 6.3 yr	Stroke Exposed = 5,252 (2.46%) Unexposed = 2,727 (2.56%)	Cox proportional hazard models	Stroke: HR 1.02 (95% CI 0.98–1.07)	Matching variables (age, sex), obesity, smoking, history of cholesterol, hypertension, diabetes, IHD, atrial fibrillation, intermittent arterial claudication, carotid stenosis, heart disease
Calabrese, 2017	Cohort	N = 43,527 Follow-up: up to 7 yr (total 64,528.2 pyr)	N = 680, 1.6%	Generalized linear models	0-90d: IRR 1.36 (1.10–1.68) 91-365d: IRR 1.18 (1.00–1.40) Baseline: 366-730days	Age, sex, race, diabetes mellitus, hypertension, atrial fibrillation, TIA, glucocorticoids.
Hosamirudsari, 2018	Case-control	Cases = 105 Controls = 105	Cases: 24/105 (22.9%) Controls: 5/105 (4.8%)	Logistic regression	OR, 5.84 (95% CI, 1.98‐ 8.23)	Age, sex, and hypertension.
Kang, 2009	Cohort	Exposed = 7760 Unexposed = 23,280 Follow-up: up to 1 year	Exposed = 133, 1.7% Unexposed = 306, 1.3%	Cox proportional hazard models	Risk of stroke during the 1-yr follow-up period: HR 1.31 (95% CI 1.06–1.60)	Age, sex, income, urbanization, geographical location, hypertension, diabetes, renal disease, CHD, hyperlipidemia, atrial fibrillation, heart failure, heart valve/myocardium disease, and/or carotid/peripheral vascular disease
Kim, 2017	Cohort	Exposed = 23,213 Unexposed = 23,213 Follow-up: up to 10 yr	Not reported	Not reported	HR 1.35 (95% CI 1.18–1.54)	Age, sex, BMI, smoking, drinking, exercise, economic class, hypertension, diabetes, dyslipidemia, angina, TIA, heart failure, atrial fibrillation, heart disease, renal disease, carotid stenosis, peripheral vascular disease, liver disease, rheumatoid disease, inflammatory bowel disease, malignancy, transplantation, HIV, depression.
Kwon, 2016	Cohort	Exposed = 77 781 Unexposed = 695755 Follow-up: up to 11 yr (total 7,770,699 years)	Crude incidence rate: 9.8/1000 py	Time-updated Cox models	18–30 yrs: HR 1.52, 95% CI 1.26–1.83 30–40 yrs: HR 1.34, 95% CI 1.19–1.51 40–50 yrs: HR 1.19, 95% CI 1.12–1.29 50–60 yrs: HR 1.12, 95% CI 1.06–1.19 60–70 yrs: HR 1.14, 95% CI 1.08–1.20 >70 yrs: HR 1.14, 95% CI 1.06–1.23	Age, gender, hypertension, hyperlipidaemia, IHD, diabetes, heart failure, peripheral vascular disease, atrial fibrillation or atrial flutter, chronic renal disease, valvular heart disease (time-updated)
Langan, 2014	SCCS	N = 6584 Follow-up (median): 12.5 yr (IQR, 8.7–17.1).	wk 1–4: n = 90 wk 5–12: n = 149 wk 13–26: n = 215 wk 27–52: n = 303	Conditional Poisson regression	wk 1–4: IR 1.63 (1.32–2.02) wk 5–12: IR 1.42 (1.21–1.68) wk 13–26: IR 1.23 (1.07–1.42) wk 27–52: IR 0.99 (0.88–1.12)	Age and time-invariant confounders
Liao, 2017	Cohort	HZ patients = 2744 Non HZ patients = 5475	Exposed = 116, 4.2% Unexposed = 186, 3.4%	Cox proportional hazard models	0-90d: HR 2.30 (95%CI 1.13–4.69) 91-365d: HR 1.05 (95%CI 0.58–1.90) 366-730d: HR 1.16 (95%CI 0.70–1.92) >730d: HR 1.18 (95%CI 0.86–1.64)	Age, sex, atrial fibrillation, CKD, COPD, diabetes mellitus, dyslipidemia, and hypertension
Lin, 2010	Cohort	Exposed = 658 Unexposed = 1974 Follow-up: up to 1 yr	Exposed = 53, 8.1% Unexposed = 33, 1.7%	Cox proportional hazard regressions	HR 4.52 (95% CI 2.45–8.33)	Age, gender, hypertension, diabetes, hyperlipidemia, CHD, chronic rheumatic heart disease, other forms of heart disease, and medication habits
Minassian, 2015	SCCS	N = 42,954 Follow-up (median): 5 yr (IQR: 4–5 yr)	Baseline: n = 32179 wk 1: n = 499 wk 2–4: n = 967 wk 5–12: n = 1841 wk 13–26: n = 2588 wk 27–52: n = 3981	Conditional Poisson regression	wk 1: IR 2.37, 95% CI 2.17–2.59 wk 2–4: IR 1.55, 95% CI 1.46–1.66 wk 5–12: IR 1.17, 95% CI 1.11–1.22 wk 13–26: IR 1.03, 95% CI 0.99–1.07 wk 27–52: IR 1.00, 95% CI 0.96–1.03	Age in 2-yr age bands and time-invariant confounders
Patterson, 2018	Cohort	Exposed = 23,339 Unexposed = 46,378 Follow-up: up to 10 yr	Exposed = 141, 6.0% Unexposed = 262, 5.6%	Multivariate Poisson models	IRR 1.40 (95%CI 0.93–2.11)	Sociodemographic and clinical factors, including smoking status and BMI
Schink, 2016	SCCS	N = 6,035 Followup time (mean): 5.6 yr	3 mo following zoster: N = 352	Log-linear Poisson model	<2 wk: 1.30 (1.00–1.68) wk 3–4: IRR 1.52 (1.20–1.91) mo 2–3: IRR 1.24 (1.08–1.42) mo 4–6: IRR 1.09 (0.97–1.24) mo 7–12: IRR 0.96 (0.87–1.06)	Age
Sreenivasan, 2013	Cohort	N = 4,503,054 Exposed: 117926 Follow-up: up to 14 years	Overall: N = 230341, 5.0%. Exposed: 4876/117926, 4.1%	Poisson regression	< 14 days since HZ: IRR 2.27 (95%CI 1.83–2.82) 14 days-1 yr: IRR 1.17 (95%CI 1.09–1.24) > 1 yr: IRR 1.05 (95%CI 1.02–1.09)	Age, sex, calendar period.
Sundström, 2015	Cohort	General population = 4,707,885 Exposed = 13296 All followed for 1-yr.	Exposed = 111 General population = unknown	Poisson regression	IRR 1.34 (95% CI 1.12–1.62)	Age and sex.
Tseng, 2011	Cohort	Not reported Follow-up: up to 4 years	Exposed = 227 Unexposed = 224	Not reported	HR 1.11 (95% CI 0.92 to 1.33)	Matching factors (age and sex), race, heart diseases, diabetes, lung, kidney, liver disease, hypertension, dementia
Yawn, 2016	Cohort	Exposed = 4478Unexposed = 16,800Follow-up (mean): 7.1 yr (range 0–28.6 yr)	EverExposed = 562, 12.6%Unexposed = 1844, 11.0%	Logistic regression	OR (95% CI):3 mo: 1.53 (1.01–2.33)6 mo: 1.28 (0.91–1.80)1 yr: 1.04 (0.79–1.36)3 yr: 1.02 (0.86–1.22)	3 mo: Age, vasculopathy, arrhythmias.6 mo: Age, vasculopathy, hypertension.1 yr: Age, vasculopathy, hypertension, CAD, dyslipidemia.3 yr: Age, gender, hypertension, CAD, dyslipidemia, depression, vasculopathy.
**CMV infection**						
Coles, 2003	Case-cohort	Stroke cases = 119 Random sub-cohort = 451 Follow-up time: up to 3 years	CMV IgG: Stroke cases: 84.9%; Random sub-cohort: 85.4% High level CMV IgG: Stroke cases: 40.3%; Random sub-cohort: 38.1%	Cox proportional hazards regression	CMV IgG: RR 0.93 (95% CI 0.46, 1.89) CMV IgG high titre: RR 0.78 (95% CI 0.49, 1.23)	Age, gender, BMI, cholesterol, triglycerides, diabetes, haemoglobin, treatment for hypertension, systolic blood pressure and smoking.
Fagerberg, 1999	Cohort	N = 152 Follow-up (median): 6.5 yr (range 0.2–7.5)	Not reported	Poisson regression	Relative Risk of High Titres of Antibodies to CMV for Stroke: RR 1.04 (95% CI 0.13–8.51)	Smoking, presence of previous cardiovascular disease, group allocation in the underlying multiple risk factor intervention study (multifactorial risk factor intervention or usual care)
González-Quijada, 2015	Case-control	Cases = 111 Controls = 523	Cases: Seropositive CMV = 98, 95.1%; High-titre IgG antibodies = 37, 35.0% Controls: Seropositive CMV = 455, 92.9%; High-titre IgG antibodies = 109, 22.2%	Logistic regression	High-titre IgG antibodies (top quartile) against CMV (OR 2.1, 95% CI 1.3 to 3.5)	Adjusted for sex, age >81 yr, hypertension, dyslipidaemia, smoking habits, diabetes, cardioembolic focus, other vascular diseases, white blood cells, and C-reactive protein.
Huang, 2012	Case-control	Cases = 200 Controls = 200	CMV DNA Cases (stroke) = 110, 55% Controls = 47, 23.5%	Logistic regression	Odds of stroke associated with CMV DNA Any stroke: OR 3.98 (95%CI 2.50–6.32)	Age, sex, BMI, hypertension and smoking
Kenina, 2010	Case-control	Cases = 102 Controls = 48	CMV seropositivity Cases = 95/102, 93%; Controls = 45/48, 94% Mean CMV IgG antibody levels (IU/ml) Cases: 6.43 ± 2.6; Controls: 5.83 ± 2.7	None	OR for CMV seropositivity: OR 0.90 (95% CI 0.22–3.66) [calculated by review authors]	None
Oliveras, 2003	Cohort	N = 403 Time from RT until stroke = 49.3 mo (SD = 25.6 mo)	Total: N = 19 (7.97%) at 10 yr follow-up. Denominator inferred to be 238 Exposed: 0/16, 0% Unexposed: 19/387, 4.9%	Chi-squared test	RR 0.60 (95% CI 0.03–10.41)^1^ [calculated by review authors]	None
Shen, 2011	Case-control	Cases = 81 Controls = 72	Cases: 40/81 (49.4%) Controls: 13/72 (17.8%)	Chi-squared test	OR 4.51 (95% CI: 2.16–9.40)	None
Smieja, 2003	Cohort	N = 3168 Follow-up (mean) = 4.5 yr	Overall: 107/3164 (3.4%)	Cox proportional hazards	HR 0.93 (95% CI 0.61, 1.42)	Age, sex, smoking status, ramipril randomization, diabetes mellitus, hypertension, and history of hypercholesterolemia
Tarnacka, 2002	Case-control	Cases: n = 56 "Old" controls: n = 53 "Young" controls: n = 57	IC Containing Anti-CMV Antibodies Cases: 41/55 (74.5%) Old controls: 11/42 (26.2%) Young controls: 23/57 (40.4%)	Not reported	Increased levels of serum CMV-specific IC were connected with increased risk of stroke incidence (odds ratio, 7.60; 95% CI, 3.21 to 17.96)^3^.	None reported
Yi, 2008	Case-control	Cases = 35 Controls = 20	CMV IE genes/proteins Cases: 21/35 (60.0%), Controls: 6/20 (30.0%) CMV L genes/proteins Cases: 7/35 (20.0%), Controls: 4/20 (20.0%)	Chi-squared tests	CMV IE genes/protein: 3.50 (1.08–11.29) CMV L genes/protein: 1.00 (0.25–3.95) [calculated by review authors—matching not accounted for]	No adjustments made—matched on age and sex
Zheng, 2016	Case-control	Controls = 300 Cases = 300 Follow-up (median): 8.4 yr	Proportion of patients with CMV DNA Cases: 38/300 (12.7%) Controls: 17/300 (5.7%)	Conditional logistic regression	OR 1.46 (95% CI, 1.00–2.14)	Matching factors (age, gender, follow-up, stage of hypertension), pulse rate, BMI, LDL-C, HDL-C, triglycerides, fasting glucose, smoking, drinking, antihypertensives, statins, antiplatelet agents, and anticoagulants
Ziemann, 2016	Cohort	N = 983 Follow-up: unclear	CMV seropositive: n = 8/618 (1%) CMV seronegative: n = 6/365 (2%)	Chi-square test	Risk ratio: 0.79 (95% CI 0.28–2.25) [calculated by review authors]	None reported
**CMV reactivation**						
Yen, 2016	Cohort	Total: N = 22,581Exposed = 439, follow-up time 6.1 yr (SD = 3.8)Unexposed = 22,142, follow-up time 4.8 yr (SD = 3.7)	Exposed: 17/439 (3.2%)Unexposed: 211/22,142 (0.7%)	Cox proportional-hazards model	HR, 3.07; 95% CI, 1.70 to 5.55	Age, sex, diabetes, CKD, hypertension, CHD, cancer, dyslipidaemia, tuberculosis infection, disseminated Mycobacterium avium complex infection, pneumonia, meningitis, Penicillium marneffei infection, toxoplasma encephalitis, candidiasis, HZ and HAART.
**HHV6 infection**						
Fullerton, 2017	Case-control	Cases = 161 Controls = 34	Cases: 2/161 (1.2%) Controls: 0/34 (0%)	Not reported	OR 1.07 (95% CI 0.05–22.7)^1^ [calculated by review authors]	No adjustments made—matched on age
**VZV infection, serologically defined**						
Asiki, 2015	Case-control	Cases = 31 Controls = 132	All participants had detectable IgG and IgM antibodies against VZV	Mann–Whitney two-sample test	Median VZV IgG (IQR) at index date Cases: 2.06 (1.45–2.42) Controls: 1.91 (1.52–2.26); P value: 0.47 Median VZV IgM (IQR) at index date Cases: 0.32 (0.19–0.43) Controls: 0.29 (0.20–0.50); P value: 0.69	No adjustments made—matched on age and sex
**VZV infection, clinically defined (varicella)**						
Askalan, 2001	Cohort	Exposed = 22 Unexposed = 48 Follow-up: up to 12 months	Exposed: 10/22 (45%) Unexposed: 8/48 (17%)	Not reported	OR 4.1 (95% CI 1.3–12.9) [calculated by review authors]	No adjustments made
Sébire, 1999	Case-control	Cases = 11 Controls = 44	Cases: 7/11 (64%) Controls: 4/44 (9%)	Fisher’s exact test	OR: 17.5 (95% CI 3.53–86.83) [Calculated by review authors]	No adjustments made—matched on age, sex and site of residence
Thomas, 2014	SCCS	Children = 60, median follow-up 6.6 yr (IQR 4.7–11.7) Adults = 500, median follow-up 14.2 yr (IQR 9.9–18.8)	Children = 49 0–6 mo: 12; 7–12 mo: 6; Unexposed period: 31 Adults = 241 0–6 mo: 20; 7–12 mo: 11; Unexposed period: 210	Conditional Poisson regression for individual database, meta-analysis for combined databases	Children (fixed effects meta-analysis) 0-6mo: IR 4.07 (95% CI 1.96–8.45) 7–12 mo: IR 2.37 (95% CI 0.93–6.06) Adults (random effects meta-analysis) 0–6 mo: IR 2.13 (95% CI 1.05–4.36) 7–12 mo: IR 1.23 (95% CI 0.66–2.30)	Age (in 5-yr bands).
**Vaccination against herpesviruses**						
Baxter, 2012	Cohort	N = 29,010 Cohort followed for 180d	N = 193, with 38 confirmed after case review by specialists (risk period of the stroke unknown)	Exact conditional method	RR = 0.91; 95% CI: 0.43–1.81	None (design accounts for within person confounding)
Kovac, 2018	RCT	Vaccinated group: 13,881 Placebo group: 14,035 Follow-up (mean): 3.9 ± 0.7 years	Vaccinated group: 0(0%) Placebo group: 0 (0%)	None	OR: 1.01 (95% CI 0.02–51.0)^1^ [Calculated by review authors]	None (randomised design accounts for confounders)
Tseng, 2012	SCCS	Days 1–14: n = 167 Days 15–28: n = 147 Days 29–42: n = 169 Days 1–42: n = 468 Follow-up: 42 days	No. of cases in risk window/control window. Days 1–14: 81/86 Days 15–28: 74/73 Days 29–42: 83/86 Days 1–42: 233/235	Conditional Poisson regression	Days 1–14: RR 0.94 (95% CI 0.70–1.28) Days 15–28: RR 1.03 (95% CI 0.74–1.42) Days 29–42: RR 0.97 (95% CI 0.71–1.30) Days 1–42: RR 0.99 (95% CI 0.83–1.19)	None (design accounts for within person confounding)
Donahue, 2009	Cohort	N = 3240473 Vaccinated: 1,142,920 Unvaccinated: 2,097,553 Follow-up: up to 13 yr (total py 17.2 million)	Vaccinated: n = 39 (0.003%) (8 occurred in 12 mo risk period following vaccination) Unvaccinated: n = 164 (0.008%)	Cox regression	adjHR (95% CI) after vaccination 0 to <1 mo: 1.1 (0.1–9.2) 1 to <3 mo: 0.7 (0.1–5.7) 3 to <6 mo: 1.3 (0.3–5.6) 6 to <9 mo: 1.3 (0.4–4.9) 9 to <12 mon: 0.4 (0.0–3.2)	Gender, calendar time, geographical site, cardiac disease, rheumatic heart disease and endocarditis, CVD, sickle cell disease, conditions predisposing to vasculopathy, coagulation abnormalities, and diseases leading to a hypercoagulable state.
MacDonald, 2018	Cohort	Vaccinated: 325,729 Unvaccinated: 43,263 Follow-up: 1 yr	Vaccinated group: 25 (0.01%) Unvaccinated group: 6 (0.01%)	Cox proportional hazards model	HR 1.6 (95% CI 0.7–3.7)	Moyamoya disease, Sickle cell disease, Congenital heart disease, Meningitis, Severe sepsis, Intracranial injury, Varicella infection, AIS history before 11 months of age.
**Multiple herpesviruses infections**		** **	** **	** **	** **	** **
Al-Ghamdi, 2012	Case- control	Cases = 20 Controls = 15	HSV-1—Cases: n = 20, 100%; controls n = 14, 93.9% CMV—Cases: n = 9,45%; controls n = 3, 20%. EBV—Cases: n = 18, 90%; controls n = 14, 93.3%.	Chi-squared test	HSV-1: OR 2.86 (95% CI 0.09–91.16)^1^ CMV: OR 3.27 (95% CI 0.70–15.29) EBV: OR 0.64 (95% CI 0.05–7.83) [calculated by review author]	No adjustments made—matched on age and sex
Elkind, 2010	Cohort	N = 1625, Median follow-up 7.6 yr (IQR: 6.4–9.0) CMV = 1388 (85.4%) HSV-1 = 1402 (86.3%) HSV-2 = 928 (57.1%)	Overall: 67 strokes (56 ischaemic)	Cox proportional hazards models	CMV IgG: HR 2.19 (95%CI 0.84–5.70) HSV-1 IgG: HR 1.35 (95%CI 0.59–3.07) HSV-2 IgG: HR 1.59 (95%CI 0.91–2.76)	Age, sex, ethnicity, education, systolic blood pressure, cholesterol level, alcohol use, smoking status, waist circumference, physical activity, and CAD
Elkind, 2016	Case-control	Cases = 326Controls = 115	Past infection:HSV-1/2: Cases: 53, 16.3%; controls 24, 20.9% CMV: Cases: 95, 29.1%; controls 42, 36.5% EBV: Cases: 176, 54.0%; controls 58, 50.4% VZV: Cases: 182, 55.8%; controls 68, 59.1%Acute Infection:HSV-1/2: Cases: 80, 24.5%; controls 19, 16.5% CMV: Cases: 18, 5.5%; controls 2, 1.7% EBV: Cases: 4, 1.2%; controls 1, 0.9% VZV: Cases: 37, 11.3%; controls 3, 2.6%	Logistic regression	Past infection:HSV-1/2: OR 0.78 (95% CI 0.45–1.35)CMV: OR 0.74 (95% CI 0.47–1.17)EBV: OR 1.26 (95% CI 0.82–1.95)VZV: OR 0.93 (95% CI 0.60–1.44)Acute Infection:HSV-1/2: OR 1.68 (95% CI 0.98–3.00)CMV: OR 2.85 (95% CI 0.79–18.2)EBV: OR 1.44 (95% CI 0.21–28.4)VZV: OR 4.43 (95% CI 1.55–18.7)	Age.*Age, Race, Residence (urban, rural, suburban), country income (low/middle or high income)
Kis, 2007	Case-control	Cases = 59 Controls = 52	CMV DNA—Cases: n = 1, 1.7%; Controls: n = 0, 0% CMV IgM—Cases: n = 0, 0%; Controls: n = 0, 0% CMV IgG—Cases: n = 26, 44.1%; Controls: 11, 21.2% HSV-1 IgA—Cases: n = 24, 40.7%; Controls: n = 8, 15.7% HSV-1 IgG—Cases: n = 23, 39.0%; Controls: n = 14, 27.4% EBV IgG—Cases: n = 22, 37.3%; Controls: n = 15, 28.8% HHV-6 IgG—Cases: n = 19, 32.2%; Controls; n = 18, 34.6%	Logistic regression	Highest tertile v. lower two tertiles CMV IgG†: OR 4.95 (95% CI 1.38–17.80) HSV-1 IgA‡: OR 3.69 (95% CI 1.47–9.21) [below calculated by review authors—unadjusted] CMV DNA: OR 2.69 (95% CI 0.11–67.53)^1^ CMV IgM: OR 0.88 (95% CI 0.01–45.2)^1^ HSV1 IgG: OR 1.73 (95% CI 0.77–3.88) EBV IgG: OR 1.46 (95% CI 0.66–3.26) HHV-6 IgG: OR 0.90 (0.41–1.98)	Age, gender, smoking, alcohol, lipid, hypertension, sedimentation rate
Li, 2005	Case-control	Cases = 47 Controls = 193	CMV: Cases: 20/47 (43%^2^); Controls: 20/193 (10%) HSV-1: Cases: 6/47 (13%^2^); Controls: 7/193 (4%) HSV-2: Cases: 7/47 (15%^2^); Controls: 22/193 (11%)	Chi-squared test	CMV: OR 6.41 (95% CI 3.05–13.44) HSV-1: OR 3.89 (95% CI 1.24–12.18) HSV-2: OR 1.36 (95% CI 0.54–3.40) [calculated by review authors]	None
Ozturk, 2013	Case-control	Cases = 72 Controls = 60	CMV: Cases: n = 71/72 (98.6%); Controls: n = 58/60 (96.7%) EBV: Cases: n = 41/72 (56.9%); Controls: n = 29/60 (48.3%)	Logistic regression	CMV: OR 2.45 (95% CI 0.22–27.68) EBV: OR 1.41 (95% CI 0.71–2.81) [calculated by review authors]	No adjustments made—matched on age
Ridker, 1998	Case-control	Cases = 643 (only 271 were stroke patients) Controls = 643	HSV-1/2: Stroke cases: n = 271 (73.6%); Controls: n = 643 (69.4%) CMV: Stroke cases: n = 271 (65.3%); Controls: n = 643 (70.2%)	Conditional logistic regression	HSV-1/2: RR 1.0 (95% CI 0.7–1.5) CMV: RR 0.67 (95% CI 0.4–1.0)	Matching factors (age, smoking, follow-up), treatment assignment, BMI, hypertension, hypercholesterolemia, diabetes, and a family history of premature atherosclerosis.
Sealy-Jefferson, 2013	Cohort	Total N = 1621. CMV: 979 (60.4%) VZV: 299 (18.4%) HSV-1: 1014 (62.6%) Folow-up: up to 10 yr	CMV: Exposed: 97 (9.9%); Unexposed: 67 (10.4%) VZV: Exposed: 36 (12.0%); Unexposed: 128 (9.7%) HSV-1: Exposed: 94 (9.3%); Unexposed: 70 (11.5%)	Discrete-time logistic regression	IgG in the 75th versus 25th percentile CMV: OR 0.81 (95% CI 0.58, 1.12) VZV: OR 0.93 (95% CI 0.71, 1.20) HSV-1: OR 0.77 (95% CI 0.56, 1.07)	Hypertension, diabetes, hyperlipidaemia, smoking, atrial fibrillation, BMI, coronary heart disease and/or peripheral artery disease, education, age and gender.
Yen, 2017	Cohort	HIV patients: N = 21,375. Mean follow-up time 4.65 yr (SD 3.36).	CMV infection: 10/311 (3.2%); No CMV infection 242/21064 (1.2%) HZ: 238/20020 (1.2%); No HZ: 14/1355 (1.0%)	Cox regression model	CMV infection: HR 2.71 (95% CI 1.34 to 5.49) HZ: HR 0.80 (95% CI 0.46 to 1.40)	Age, sex, diabetes, chronic kidney disease, hypertension, coronary heart disease, cancer, dyslipidemia, and systemic lupus erythematosus and HAART.

Abbreviations: RCT = randomised controlled trial, SCCS = self-controlled case series, RR = risk (or rate) ratio, CI = confidence interval, transient ischaemic attack = TIA, COPD = chronic obstructive pulmonary disorder, CKD = chronic kidney disease, HZ = herpes zoster, HZO = herpes zoster opthalmicus, ESRD = End-stage renal disease, CT = computerised tomography, MRI = magnetic resonance imaging, yr = year, mo = mo, wk = wk, pyr = person years

^1^Due to zero events in specific cells, 0.5 was added to all cells to calculate an effect estimate.

^2^Percentages in paper recalculated due to assumed rounding error

^3^Unclear which controls were used in the calculation of the effect estimate

**Table 3:** Risk of bias summary showing judgements about each risk of bias domain.

First author, publication yr	Confounding	Selection of participants	Misclassification of variables	Bias due to missing data	Reverse Causation
*VZV reactivation—Herpes zoster*			** **		
Breuer, 2014	•	•	•	•	•
Calabrese, 2017	▪	•	•	•	•
Hosamirudsari, 2018	▪	**◊**	**◊**	•	•
Kang, 2009	•	•	•	•	•
Kim, 2017	◊	•	•	•	•
Kwon, 2016	**◊**	•	•	•	•
Langan, 2014	•	•	•	•	•
Liao, 2017	▪	•	•	•	•
Lin, 2010	•	•	•	•	•
Minassian, 2015	•	•	•	•	•
Patterson, 2018	**◊**	•	•	•	•
Schink, 2016	•	•	•	•	•
Sreenivasan, 2013	▪	•	•	•	•
Sundström, 2015	**◊**	**○**	•	•	•
Tseng, 2011	**○**	•	•	•	•
Yawn, 2016	•	•	•	•	•
*CMV infection*					
Coles, 2003	▪	•	•	•	•
Fagerberg, 1999	**◊**	▪	•	•	•
González-Quijada, 2015	▪	**◊**	•	•	**◊**
Huang, 2012	•	▪	•	•	**◊**
Kenina, 2010	**◊**	**◊**	•	•	**◊**
Oliveras, 2003	**◊**	**○**	•	•	•
Shen, 2011	**◊**	**◊**	•	•	**◊**
Smieja, 2003	▪	•	•	**○**	•
Tarnacka, 2002	**◊**	**◊**	•	•	**◊**
Yi, 2008	**◊**	**◊**	•	•	**◊**
Zheng, 2016	•	•	•	•	•
Ziemann, 2016	**◊**	▪	▪	•	•
*CMV reactivation*					
Yen, 2016	•	•	•	•	•
*HHV 6 infection*					
Fullerton, 2017	**◊**	▪	•	•	**◊**
*VZV infection*, *serologically defined*					
Asiki, 2015	▪	**◊**	▪	•	•
*VZV infection*, *clinically defined (varicella)*			** **		
Askalan, 2001	**◊**	**◊**	**◊**	•	•
Sébire, 1999	▪	**◊**	•	•	•
Thomas, 2014	•	•	•	•	•
*Vaccination against herpesviruses (e*.*g*. *zostavax vaccine)*					
Baxter, 2012	•	•	•	•	•
Kovac, 2018	•	•	•	•	•
Tseng, 2012	•	•	•	•	•
Donahue, 2009	•	•	•	•	•
MacDonaled, 2018	**○**	•	•	•	•
*Multiple herpesviruses infections*					
Al-Ghamdi, 2012 (HSV-1, CMV, EBV)	**◊**	**◊**	•	•	**○**
Elkind, 2010 (CMV, HSV1 and HSV2)	•	•	▪	**○**	•
Elkind, 2016 (HSV1/2, CMV, EBV, VZV)	•	▪	•	•	**◊**
Kis, 2007 (HSV-1, CMV, EBV and HHV-6)	▪	▪	•	•	**◊**
Li, 2005 (CMV, HSV1 and HSV2)	**◊**	**◊**	•	•	**◊**
Ozturk, 2013 (CMV, EBV)	▪	**◊**	•	•	**◊**
Ridker, 1998 (CMV, HSV1/2)	•	**◊**	•	**○**	•
Sealy-Jefferson, 2013 (CMV, HSV1 and VZV)	•	•	**◊**	•	•
Yen, 2017 (HZ and CMV disease)	•	•	•	•	•

Key

◊ High risk

▪ Moderate risk

• Low risk

○ Unclear risk

**Table 4:** Assessment of quality of evidence for outcomes.

**Quality assessment**							**Quality**
**№ of studies**	**Study design**	**Risk of bias**	**Inconsistency**	**Indirectness**	**Imprecision**	**Other considerations**	
**VZV: reactivation (herpes zoster)**							
17	observational studies	not serious	not serious ^a^	not serious	serious ^b^	strong association	⨁⨁⨁◯
						dose response gradient	MODERATE
**CMV: past infection**							
14	observational studies	serious ^c^	not serious ^d^	not serious	serious ^b^	none	⨁◯◯◯
							VERY LOW
**CMV: recent infection/reactivation**							
11	observational studies	serious ^c^	serious ^e^	not serious	serious ^b^	strong association	⨁◯◯◯
							VERY LOW
**HHV6: infection/reactivation**							
1	observational studies	very serious ^j^	not serious	not serious	very serious ^b^	none	⨁◯◯◯
							VERY LOW
**EBV: past infection**							
4	observational studies	serious ^f^	not serious ^g^	not serious	serious ^b^	none	⨁◯◯◯
							VERY LOW
**EBV: recent infection/reactivation**							
1	observational studies	very serious ^h^	not serious ^g^	not serious	very serious ^b^	none	⨁◯◯◯
							VERY LOW
**HSV-1: past infection**							
4	observational studies	not serious ^i^	serious ^e^	not serious	serious ^b^	none	⨁◯◯◯
							VERY LOW
**HSV-1: recent infection/reactivation**							
2	observational studies	serious ^h^	serious ^e^	not serious	serious ^b^	strong association	⨁◯◯◯
							VERY LOW
**HSV-2: past infection**							
1	observational studies	not serious	not serious	not serious	serious ^b^	none	⨁◯◯◯
							VERY LOW
**HSV-2: recent infection/reactivation**							
1	observational studies	very serious ^j^	not serious	not serious	serious ^b^	none	⨁◯◯◯
							VERY LOW
**HSV-1/2: past infection**							
2	observational studies	serious ^k^	not serious ^g^	not serious	serious ^b^	none	⨁◯◯◯
							VERY LOW
**HSV-1/2: recent infection/ reactivation**							
1	observational studies	serious ^k^	not serious	not serious	serious ^b^	none	⨁◯◯◯
							VERY LOW
**VZV infection, serologically defined: past infection**							
2	observational studies	serious ^k^	not serious ^g^	not serious	not serious	none	⨁◯◯◯
							VERY LOW
**VZV infection, serologically defined: recent infection/ reactivation**							
1	observational studies	serious ^k^	not serious	not serious	serious ^b^	very strong association	⨁⨁◯◯
							LOW
**VZV infection, clinically defined (varicella—adults)**							
1	observational studies	not serious	not serious	not serious	serious ^b^	strong association	⨁⨁◯◯
							LOW
**VZV infection, clinically defined (varicella—children)**							
3	observational studies	serious ^l^	not serious ^g^	not serious	serious ^b^	very strong association	⨁⨁⨁◯
						dose response gradient	MODERATE
**Varicella vaccination**							
2	observational studies	not serious	not serious	not serious	very serious ^b^	none	⨁◯◯◯
							VERY LOW
**Herpes zoster vaccination**							
3	observational studies	not serious	not serious ^g^	not serious	not serious	none	⨁⨁◯◯
							LOW

Explanations

a. None of meta-analyses suffered from considerable heterogeneity and trend of effects were very clear across studies using different study designs.

b. Wide confidence interval(s)

c. <50% studies have two or more domains at high risk of bias and contribute <50% weight to the meta-analysis

d. Some point estimates across studies in different directions, some overlap of confidence intervals, where meta-analyses were possible I^2^ statistic indicates substantial heterogeneity (although subgroup analyses demonstrated heterogeneity was driven by a single study).

e. Some variance of point estimates across studies (yet the majority are in the in same direction), some overlap of confidence intervals, where meta-analyses were possible I^2^ statistic indicates statistical heterogeneity (yet only very few studies included in meta-analyses).

f. ≥50% studies have two or more domains at high risk of bias, however contribute <50% weight to the meta-analysis

g. Some variance of point estimates across studies, confidence intervals overlap, no statistical evidence of heterogeneity

h. Study/studies suffered from two or more domains at high risk of bias.

i. 1/4 studies suffered from two domains at high risk of bias; this study only contributed 5% weight to the meta-analyses.

j. Study may have suffered from three domains at high risk of bias: confounding, reverse causality, and selection bias

k. Study/studies had one domain at high risk of bias.

l. 2/3 studies suffered from 2 or more domains at high risk of bias, however one study had a very low risk of bias

17 studies assessed the association between zoster and stroke (1 case-control study,[21] 13 cohort studies[22–34] and 3 SCCS[35–37]) (Table 1). Ten were based in the US or Europe and six in Asia and one in the Middle East; all studies used routinely collected medical records. Two studies involved an immunosuppressed population. 8/17 studies were considered at low-risk of bias in all domains.

Zoster was associated with a 1.5-fold increased stroke risk four weeks following onset (summary estimate: 1.55, 95%CI 1.46–1.65), with the risk decreasing to baseline after around one year (Fig 2). Removing three studies at high-risk of bias eliminated statistical heterogeneity in cohort studies with “Over 1 year follow-up” (I^2^<0.01%, see S1 Table). There were no SCCS at high-risk of bias. There was moderate quality evidence of an increased risk of stroke following zoster, with evidence upgraded due to some strong associations and a clear dose-response gradient over time.

Two studies reported an increased risk of TIA following zoster. The first showed over 50% increased risk (IRR1.56, 95%CI:1.13–2.15) over a maximum of 10 years follow-up[34] and the second around 15% increased risk (HR1.15, 95%CI:1.09–1.21) during a median follow-up of 6.3 years.[22] Only a single SCCS study assessed the effect of zoster vaccination on stroke risk, using Medicare claims data; this study found no evidence that zoster vaccination attenuated stroke risk, however only 3% of study participants were vaccinated which limited the study’s ability to detect an effect.[35]

Results can be found in S1, S2, S3, S4 and S5 Figs. Ophthalmic zoster was associated with increased risk of stroke, of a larger magnitude than zoster at any site. The pooled estimate for stroke up to 4 weeks following ophthalmic zoster in SCCSs was 1.77 (95%CI:1.53–2.05), compared to 1.55 (95%CI:1.46–1.65) following any zoster (S1 Fig). Another study found the elevated risk of stroke among rheumatoid arthritis patients experiencing zoster was greatest in those patients with a neurological complication.[33] Antiviral agents appeared to attenuate stroke risk in two out of three studies, though the confidence intervals for effect estimates for zoster patients given and not given antivirals overlapped (S2 Fig). In one SCCS study, in the first four weeks following zoster there appeared to be no evidence of an increased risk of stroke among those given antivirals (IRR1.23, 95%CI:0.89–1.70), whilst for those not given antivirals there was an association (IRR2.14, 95%CI:1.62–2.83). A larger effect of zoster on stroke risk was seen in people aged below 40 years (S3 Fig); there was no difference of zoster on stroke risk by gender (S4 Fig); and little difference in stroke risk by stroke type (ischaemic versus haemorrhagic), except in one cohort study from Taiwan[24] where the magnitude of association was greater for haemorrhagic stroke (S5 Fig).

CMV infection, defined largely using laboratory criteria, was investigated in 22 studies[9, 30, 38–57] using data from a variety of settings including electronic healthcare records, survey data and trial data (Table 1).

Among studies assessing CMV infection (past or recent), 19/22 studies had a least one domain at high-risk of bias, including: confounding (ten studies had no age-adjustment) and reverse causation (10 studies recorded CMV following stroke).

14 studies investigated past CMV infection and stroke risk (Fig 3); IgG seropositivity and/or high titre IgG antibodies were investigated. IgG seropositivity was not associated with stroke when combining six case-control studies (summary estimate:1.40, 95%CI:0.67–2.96; I^2^ = 78.8%) nor in cohort studies (summary estimate:1.01,95%CI:0.73–1.39, I^2^<0.001%). While having a high IgG titre compared to a low titre was associated with stroke when combining two case-control studies (summary estimate:2.61,95%CI:1.26–5.43, I^2^ = 33.4%) it was not associated with stroke when pooling three cohort studies (summary estimate:0.80,95%CI:0.62–1.05, I^2^<0.001%).

Recent CMV infection or reactivation was investigated in 11 case-control studies (Fig 3), using a variety of exposure definitions. In a meta-analysis of two studies, IgM seropositivity was associated with increased stroke risk (summary estimate:5.53,95%CI:2.83–10.81, I^2^<0.001%). When pooling three studies, CMV DNA was also associated with increased stroke risk (summary estimate:2.34,95%CI:0.95–5.74, I^2^ = 81.4%). In two of three studies among immunosuppressed patients, clinical CMV reactivation was associated with around 3-fold increased risk of stroke.

There was very low-quality evidence suggesting there is no association between past infection with CMV and stroke and an increased risk of stroke following recent infection/reactivation with CMV.

CMV was the only outcome for which sufficient studies were available to assess publication bias; there was no evidence of publication bias (see S6 Fig).

One case-control study assessed the association between HHV-6 and stroke; no association was found.[58] Four case-control studies examined the association between EBV and stroke (Table 1);[50, 51, 53, 55] three were hospital-based among older adults and one a multi-country study among children (under 18 years). All studies were small (N<500) and at high-risk of bias.

There was no evidence that past infection (IgG seropositivity) was associated with stroke risk, when combining data from three studies (summary estimate: 1.28, 95%CI:0.89–1.84; I^2^<0.001) (Fig 4). The study among children found no evidence that recent infection/reactivation of EBV (measured from IgM seropositivity) was associated with stroke risk (OR 1.44, 95% CI 0.12–16.75).

There was very low quality evidence of no association between past infection and an increased risk following recent infection/reactivation with EBV and stroke; the quality of evidence was downgraded due to high-risk of bias and imprecise estimates.

Associations between HSV-1 or HSV-2 and stroke risk were explored in seven studies[50–54, 56, 57] (Table 1) using population survey data and an RCT, and data from a hospital setting. A high-risk of bias was identified in all seven studies. No clear patterns were observed, although there was some indication that recent HSV1 infection/reactivation (IgM seropositivity or IgA high titre) was associated with increased stroke risk.

Two case-control studies[51, 59] and one US-community based cohort study[57] assessed the effect of serologically-defined VZV infection on stroke risk (Table 1). Past infection (IgG seropositivity or high titre) was not associated with stroke risk in two studies (Fig 4); quality of evidence was graded very low due to a high-risk of bias. However, a multi-country case-control study among children (under 18 years) found recent infection/reactivation (IgM seropositivity) was associated with increased stroke risk; quality of evidence was graded as low, because although there was a high-risk of bias, the association was very strong.

Varicella and the risk of stroke among children was assessed in three studies from Canada and Europe;[60–62] a high-risk of bias was identified in 2/3 studies. Different study designs and time periods during which stroke was recorded were used, therefore estimates were not pooled. However, each study found varicella was associated with a greater risk of stroke within a year from diagnosis. Because of the dose-response gradient over time and very strong associations observed, the evidence was classified as moderate quality.

The SCCS study also assessed the association among adults; an increased risk of stroke within 6-months of varicella was found (IR2.13, 95%CI:1.05–4.34). Although this association was strong, the confidence interval was wide, thus the evidence was graded low quality.

Five studies evaluated the short-term effect of VZV vaccination on stroke risk, by comparing vaccinated with unvaccinated people (or person time in the same individuals). One was a multi-country RCT[63] and the others used Canadian or US electronic healthcare records (Table 1);[64–67] these studies were at very low-risk of bias. No decreased risk of stroke in those vaccinated against varicella or zoster was noted (Fig 4); evidence across studies was graded very low and low quality for varicella and zoster vaccination, respectively.

One small (N = 111) case-control study among older hospitalised patients found no association between HHV-6 IgG seropositivity and stroke (Table 1), in unadjusted analysis (OR 0.90, 95%CI:0.41–1.98).[53] No studies assessed herpesvirus-7 or 8.

## Discussion

Our review identified 48 studies assessing the association between infection with or reactivation of herpesviruses and risk of stroke. Consistent with previous reviews, there was moderate quality evidence that zoster was associated with a short-term increased risk of stroke, and that increased risk was greatest shortly after zoster (decreasing to baseline by around one year). Some evidence suggested the risk was greater among ophthalmic zoster patients, younger age groups and patients not prescribed antivirals. Moderate quality evidence suggests varicella was associated with increased stroke risk in children. Similar to findings for VZV, there may also be an increased stroke risk with recent CMV and HSV infection/reactivation, however the evidence was very low quality. Finally, there might be an increased stroke risk associated with recent CMV infection or reactivation based on studies carried out in immunosuppressed populations.

Two main pathophysiological mechanisms are proposed by which herpesviruses may increase stroke risk. Systemic infection with, or reactivation of, herpesviruses induces acute inflammation,[2] which may lead to endothelial dysfunction accompanied by disruption of atheromatous plaques and hypercoagulability.[68] This biological hypothesis is consistent with our finding that latent herpesvirus infection (that is, presence of viral DNA in host cells without producing infectious viral particles)[69] does not appear to increase stroke risk, as latent infection does not cause acute inflammation in host cells. Herpesviruses may also directly invade cerebral arteries, producing vasculopathy, leading to increased stroke risk;[70] this could explain why younger individuals, normally free from traditional vascular risk factors, were at higher risk of stroke following a recent infection/reactivation of VZV. VZV is the only virus with clear evidence of virus DNA in cerebral arteries; the stronger association between ophthalmic zoster and stroke also supports this hypothesis. CMV is associated with vasculopathy in immunocompromised patients, however the mechanism, and the risk in immunocompetent subjects are unclear.[71]

A larger effect of zoster on stroke risk was identified in people aged below 40 years. This has also been reported in a Korean-based cohort study. However, the absolute risk of stroke is low in younger ages, so a large relative effect may be small in absolute terms. This finding, together with the clinical efficacy of the currently available zoster vaccine becoming limited beyond 5–8 years,[72, 73] means vaccinating younger age groups may not be cost-effective.

This is the first study to systematically review the literature on all eight human herpesviruses as stroke risk factors and the results are broadly in-line with previous review assessing individual herpesviruses and cardiovascular disease (including stroke risk).[3–8, 10] Strengths included: following a pre-specified protocol; undertaking a comprehensive search; using articles published in any language; and carrying out a complete risk of bias assessment for each study and an assessment of the accumulated evidence using GRADE. Most studies ascertained stroke from pre-existing health care records (n = 33/41), potentially leading to similar stroke definitions across studies. A further strength of this review is that it not only included studies of clinically apparent herpesviruses reactivation, but subclinical reactivation. A further strength of this review is that it not only included studies of clinically apparent herpesviruses reactivation, but subclinical reactivation. It is possible that those with clinical manifestations of reactivated infection (e.g. zoster), or those who are immunosuppressed (as in some CMV studies), may have higher viral titres which plausibly could affect the risk of short term triggering of stroke.

However, limitations included having little data from low-income countries, which make up around 75% of stroke deaths worldwide;[74] whether different populations have different susceptibilities to stroke following herpesvirus infections is unclear. Some meta-analyses combined very few studies, limiting the strength of our pooled results. Overall, the quality of evidence for CMV, EBV and HSV was low or very low.

The studies of VZV, particularly zoster, were well-powered to assess the association between VZV and stroke and rarely suffered from a high-risk of bias; however, subgroup analyses were underpowered, limiting confidence in the findings. Studies of the other herpesviruses (CMV, EBV and HSV) had more limitations; many had small sample sizes, inadequate adjustment for confounders In addition to this, the majority of non-VZV studies relied on laboratory, rather than clinical, identification of possible recent infection or reactivation. The strength of the evidence for zoster and stroke risk lies in the studies all using clear clinical diagnoses of reactivated VZV, which was recorded prior to stroke. In contrast to VZV infection or reactivation which presents with clear clinical symptoms, other herpesviruses may reactivate without any clinical symptoms. Studies that measured markers of infection after stroke may suffer from reverse causality (all but one cases-control study–see Table 3) herpesvirus exposures were defined following stroke and stroke may trigger stress, leading to detection of herpesviruses reactivation after 24 hours (and blood samples were rarely taken immediately after stroke). This may explain why CMV IgG high (versus low) titre was associated with increased stroke risk in most case-control studies,[18] but not cohort studies (in which CMV antibodies were recorded prior to stroke). However, most case-control studies used hospital-based controls, so any stress associated with hospitalisation itself may affect cases and controls equally.

In terms of future research, high-powered cohort or SCCS studies assessing the association between recent infection with, and reactivation of, herpesviruses (aside from VZV), ideally collecting serology samples regularly during follow-up are needed. Furthermore, as zoster vaccination uptake increases, better-powered studies could confirm our findings that vaccination is not associated with a short-term increased stroke risk, and establish whether the vaccine reduces the long-term risk of stroke.

In terms of clinical practice, this review indicated that antivirals might attenuate stroke risk among zoster patients. As patients with more severe zoster are more likely to get antivirals, and also potentially more likely to have a stroke, this might have led to underestimation of their effect through confounding by indication. Antiviral drugs shorten zoster healing time and reduce pain severity[75] therefore these drugs may plausibly reduce stroke risk, by reducing inflammation. Antivirals for zoster are under-prescribed in UK primary care[76] and this review strengthens the argument for better adherence to prescribing guidelines.

Our review highlights that we have a good understanding of a short-term increased stroke risk following VZV infection and reactivation. It also suggests infection and reactivation of other herpesviruses may increase stroke risk, yet better evidence is required. Herpesviruses are common, therefore improved understanding of whether they increase the risk of stroke could provide additional strategies for stroke prevention.

## Supporting information

S1 Appendix Search terms. (PDF)

S2 Appendix Changes to the original protocol. (DOCX)

S3 Appendix Extracted data items. (DOCX)

S4 Appendix Grade assessment of quality: Down/ up-grading reasons. (DOCX)

S5 Appendix Reference list for selected studies. (DOCX)

S1 Fig Effect of clinically diagnosed ophthalmic zoster on stroke risk, by study design and length of follow-up. (DOCX)

S2 Fig Effect of zoster on stroke risk by length of follow-up and antiviral use during acute zoster. (DOCX)

S3 Fig Effect of zoster on stroke risk by length of follow-up and age group. (DOCX)

S4 Fig Effect of zoster on stroke risk by length of follow-up and gender. (DOCX)

S5 Fig Effect of zoster on stroke risk by length of follow-up and type of stroke. (DOCX)

S6 Fig Assessment of publication bias for CMV IgG seropositivity as a risk factor for stroke. (DOCX)

S1 Table Exploring statistical heterogeneity identified in meta-analyses. (DOCX)

S2 Table Risk of bias. (PDF)
