# Supplementary material for: Association of herpesviruses and stroke: Systematic review and meta-analysis
Source: PLoS One. 2018 Nov 21;13(11):e0206163. doi: 10.1371/journal.pone.0206163 (PMC6248930; doi:10.1371/journal.pone.0206163)
Supplement: S1 Appendix — (PDF) [file pone.0206163.s001.pdf]

MEDLINE (OVID) search strategy [1946 to February Week 1 2017]

1 exp Herpes simplex
2 exp Herpes simplex virus vaccines
3 exp encephalitis, herpes simplex
4 exp Herpesvirus 1, Human
5 cold sore$.ti,ab.
6 exp Herpesvirus 2, Human
7 (genit$ herpes$ or genit$ sores).ti,ab.
8 exp Chickenpox
9 exp Chickenpox vaccine
10 exp Herpes zoster
11 exp Neuralgia, postherpetic
12 exp Herpesvirus 3, Human
13 exp Encephalitis, varicella zoster
14 (varicella or chickenpox or chicken pox or shingles or VZV or zoster).ti,ab.
15 exp Cytomegalovirus
16 exp Cytomegalovirus vaccines
17 exp Cytomegalovirus infections
18 (CMV or cytomegalovirus).ti,ab.
19 exp Herpesvirus 6, Human
20 Roseolovirus Infections/
21 Exanthema Subitum/
22 (B lymphotropic virus$ or roseola or sixth disease or exanthema subitum or exanthem criticum or Roseolovirus or pseudorubella or three?day fever).ti,ab.
23 exp Herpesvirus 7, Human
24 exp Epstein-Barr virus infections
25 exp Epstein-Barr virus
26 exp Herpesvirus 4, Human
27 (EBV or epstein-barr or burkitt adj5 lymphoma$ or glandular fever or infectious mono$ or mononucleosis or hair$ leukoplak$ or OHL)
28 exp Herpesvirus 8, Human
29 Sarcoma, Kaposi/
30 Lymphoma, Primary Effusion/
31 (kaposi$ sarcoma$ or Primary effusion adj2 lymphoma$ or body cavity adj2 lymphoma$).ti,ab.
32 ((HHV adj1 ("1" or "2" or "3" or "4" or "5" or "6" or "7" or "8")) or (HHV?1 or HHV?2 or HHV?3 or HHV?4 or HHV?5 or HHV?6 or HHV?7 or HHV?8)).ti,ab.
33 (HSV?1 or HSV 1 or HSV?2 or HSV 2).ti,ab.
34 herpes$.ti, ab.
35 exp acyclovir
36 ganciclovir/ or foscarnet/ or Idoxuridine/ or Trifluridine/
37 (ac?clovir or Zovirax or valac?clovir or valtrex or famc?clovir or famvir or penc?clovir or ganc?clovir or cidofovir or foscarnet$ or valganc?clovir or lubocavir or brivudin or Docosanol or Sorivudine or Idoxuridine or Trifluridine).ti,ab
38 or 1/37
39 exp stroke/
40 exp "Intracranial Embolism and Thrombosis"/
41 exp intracranial hemorrhages/
42 aneurysm, ruptured/ and exp brain/
43 Ischemic Attack, Transient/
44 (stroke or cva or (cerebrovasc$ AND (disease or event or accident or attack or injury))).ti,ab.
45 ((brain$ or cerebr$ or cerebell$ or cortical or vertebrobasilar or hemispher$ or intracran$ or intracerebral or infratentorial or supratentorial or MCA or anterior circulation or posterior circulation or basal ganglia) adj5 (isch?emi$ or infarct$)).ti,ab.
46 ((lacunar or cortical) adj5 infarct$).ti,ab.
47 ((intracran$ or intracerebral) adj3 (thrombo$ or thrombus$ or embol$)).ti,ab.
48 SAH.ti,ab.
49 ((brain$ or cerebr$ or cerebell$ or intracerebral or intracran$ or parenchymal or intraventricular or infratentorial or supratentorial or basal gangli$ or subarachnoid or putaminal or putamen or posterior fossa) adj5 (haemorrhage$ or hemorrhage$ or bleed$ or rupture$ aneurysm)).ti,ab.
50 ((tia$1 or transi$) adj3 (isch?emia$ attack or brain isch?emia$ or cerebral isch?emia$ or CVA$ or cerebral vasc$ or
51 or 39/50
52 38 and 51

Embase (OVID) search strategy [1947 to 2017 February 08]

1 exp herpes simplex/
2 exp herpes simplex virus/
3 herpes simplex vaccine/
4 cold sore$.ti,ab.
5 (genit$ herpes$ or genit$ sores).ti,ab.
6 Chickenpox/
7 Chickenpox vaccine/
8 exp herpes zoster/
9 exp Varicella zoster virus/
10 postherpetic neuralgia/
11 (varicella or chickenpox or chicken pox or shingles or VZV or zoster).ti,ab.
12 exp Human cytomegalovirus/
13 Cytomegalovirus vaccine/
14 exp Cytomegalovirus infection/
15 (CMV or cytomegalovirus).ti,ab.
16 Human herpesvirus 6/
17 Exanthema Subitum/
18 (B lymphotropic virus$ or roseola or sixth disease or exanthema subitum or exanthem criticum or Roseolovirus or pseudorubella or three?day fever).ti,ab.
19 Human herpesvirus 7/
20 exp Epstein-Barr virus infection/
21 Epstein-Barr virus/
22 Mononucleosis/
23 (EBV or epstein-barr or burkitt adj5 lymphoma$ or glandular fever or infectious mono$ or mononucleosis or hair$ leukoplak$ or OHL).ti,ab.
24 Herpesvirus 8, Human/
25 Kaposi sarcoma/
26 Primary effusion lymphoma/
27 (kaposi$ sarcoma$ or Primary effusion adj2 lymphoma$ or body cavity adj2 lymphoma$).ti,ab.

28 ((HHV adj1 ("1" or "2" or "3" or "4" or "5" or "6" or "7" or "8")) or (HHV?1 or HHV?2 or HHV?3 or HHV?4 or HHV?5 or HHV?6 or HHV?7 or HHV?8)).ti,ab.
29 (HSV?1 or HSV 1 or HSV?2 or HSV 2).ti,ab.
30 herpes$.ti, ab.
31 exp acyclovir/
32 ganciclovir/ or foscarnet/ or Idoxuridine/ or Trifluridine/
33 (ac?clovir or Zovirax or valac?clovir or valtrex or famc?clovir or famvir or penc?clovir or ganc?clovir or cidofovir or foscarnet$ or valganc?clovir or lubocavir or brivudin or Docosanol or Sorivudine or Idoxuridine or Trifluridine).ti,ab
34 or 1/33
35 exp basal ganglion hemorrhage
36 exp brain hematoma/
37 exp brain hemorrhage/
38 exp brain infarction/
39 exp cerebrovascular accident
40 brain artery aneurysm rupture/
41 aneurysm rupture/ AND exp brain/
42 exp occlusive cerebrovascular disease/
43 transient ischemic attack/
44 brain embolism/
45 (stroke or cva or (cerebrovasc$ AND (disease or event or accident or attack or injury))).ti,ab.

46 ((brain$ or cerebr$ or cerebell$ or cortical or vertebrobasilar or hemispher$ or intracran$ or intracerebral or infratentorial or supratentorial or MCA or anterior circulation or posterior circulation or basal ganglia) adj5 (isch?emi$ or infarct$)).ti,ab.
47 (((lacunar or cortical) adj5 infarct$)).ti,ab.
48 ((intracran$ or intracerebral) adj3 (thrombo$ or thrombus$ or embol$)).ti,ab.
49 SAH.ti,ab.
50 ((brain$ or cerebr$ or cerebell$ or intracerebral or intracran$ or parenchymal or intraventricular or infratentorial or supratentorial or basal gangli$ or subarachnoid or putaminal or putamen or posterior fossa) adj5 (haemorrhage$ or hemorrhage$ or bleed$ or rupture$ aneurysm)).ti,ab.

51 ((tia$1 or transi$) adj3 (isch?emia$ attack or brain isch?emia$ or cerebral isch?emia$ or CVA$ or cerebral vasc$ or cerebrovasc$)).ti,ab.
52 or 35/51
53 34 AND 52

Global Health (OVID) search strategy [1910 to 2017 Week 04]

1 exp Human herpesviruses/
2 exp Human herpesvirus 1/
3 exp Human herpesvirus 2/
4 exp Herpes simplex viruses/
5 cold sore$.ti,ab.
6 (genit$ herpes$ or genit$ sores).ti,ab.
7 exp Varicella/
8 exp Human herpesvirus 3/
9 exp Herpes zoster/
10 (varicella or chickenpox or chicken pox or shingles or VZV or zoster).ti,ab.
11 exp Human herpesvirus 5/
12 (CMV or cytomegalovirus).ti,ab.
13 exp Human herpesvirus 6/
14 exp Roseolovirus/
15 Exanthema subitum/
16 (B lymphotropic virus$ or roseola or sixth disease or exanthema subitum or exanthem criticum or Roseolovirus or pseudorubella or three?day fever).ti,ab.
17 exp Human herpesvirus 4/
18 (EBV or epstein-barr or burkitt adj5 lymphoma$ or glandular fever or infectious mono$ or mononucleosis or hair$ leukoplak$ or OHL).ti,ab.
19 exp Human herpesvirus 8/
20 (kaposi$ sarcoma$ or Primary effusion adj2 lymphoma$ or body cavity adj2 lymphoma$).ti,ab.

21 Kaposi's sarcoma/
22 ((HHV adj1 ("1" or "2" or "3" or "4" or "5" or "6" or "7" or "8")) or (HHV?1 or HHV?2 or HHV?3 or HHV?4 or HHV?5 or HHV?6 or HHV?7 or HHV?8)).ti,ab.
23 (HSV?1 or HSV 1 or HSV?2 or HSV 2).ti,ab.
24 herpes$.ti, ab.
25 exp Aciclovir/
26 aciclovir/ or cidofovir/ or famciclovir/ or foscarnet sodium/ or ganciclovir/ or penciclovir/ or valaciclovir/
27 (ac?clovir or Zovirax or valac?clovir or valtrex or famc?clovir or famvir or penc?clovir or ganc?clovir or cidofovir or foscarnet$ or valganc?clovir or lubocavir or brivudin or Docosanol or Sorivudine or Idoxuridine or Trifluridine).ti,ab
28 or 1/27
29 stroke/
30 exp brain/ and exp haemorrhage/
31 (stroke or cva or (cerebrovasc$ AND (disease or event or accident or attack or injury))).ti,ab.

32 ((brain$ or cerebr$ or cerebell$ or cortical or vertebrobasilar or hemispher$ or intracran$ or intracerebral or infratentorial or supratentorial or MCA or anterior circulation or posterior circulation or basal ganglia) adj5 (isch?emi$ or infarct$)).ti,ab.
33 (((lacunar or cortical) adj5 infarct$)).ti,ab.
34 ((intracran$ or intracerebral) adj3 (thrombo$ or thrombus$ or embol$)).ti,ab.
35 SAH.ti,ab.
36 ((brain$ or cerebr$ or cerebell$ or intracerebral or intracran$ or parenchymal or intraventricular or infratentorial or supratentorial or basal gangli$ or subarachnoid or putaminal or putamen or posterior fossa) adj5 (haemorrhage$ or hemorrhage$ or bleed$ or rupture$ aneurysm)).ti,ab.

37 ((tia$1 or transi$) adj3 (isch?emia$ attack or brain isch?emia$ or cerebral isch?emia$ or CVA$ or cerebral vasc$ or cerebrovasc$)).ti,ab.
38 or 29/37
39 28 and 38

The Cochrane Library search strategy [inception to February 9th 2017]

1 (herpes*):ti,ab,kw
2 (((HHV NEAR/1 ("1" or "2" or "3" or "4" or "5" or "6" or "7" or "8")) or (HHV?1 or HHV?2 or HHV?3 or HHV?4 or HHV?5 or HHV?6 or HHV?7 or HHV?8))):ti,ab,kw
3 (HSV1 or "HSV 1" or HSV2 or "HSV 2"):ti,ab,kw
4 (ac?clovir or Zovirax or valac?clovir or valtrex or famc?clovir or famvir or penc?clovir or ganc?clovir or cidofovir or foscarnet$ or valganc?clovir or lubocavir or brivudin or Docosanol or Sorivudine or Idoxuridine or Trifluridine):ti,ab,kw
5 (kaposi* sarcoma* or Primary effusion lymphoma* or body cavity lymphoma*):ti,ab,kw
6 (EBV or epstein-barr or burkitt NEAR/5 lymphoma* or glandular fever or infectious mono* or mononucleosis or hair* leukoplak* or OHL):ti,ab,kw
7 (B lymphotropic virus* or roseola or sixth disease or exanthema subitum or exanthem criticum or Roseolovirus or pseudorubella or three?day fever):ti,ab,kw
8 (CMV or cytomegalovirus):ti,ab,kw
9 (varicella or chickenpox or chicken pox or shingles or VZV or zoster):ti,ab,kw
10 ("genit* herpes*" or "genit* sores"):ti,ab,kw
11 (cold sore*):ti,ab,kw
12 #1 or #2 or #3 or #4 or #5 or #6 or #7 or #8 or #9 or #10 or #11
13 (stroke or cva or (cerebrovasc* AND (disease or event or accident or attack or injury))):ti,ab,kw

14 ((brain* or cerebr* or cerebell* or cortical or vertebrobasilar or hemispher* or intracran* or intracerebral or infratentorial or supratentorial or MCA or anterior circulation or posterior circulation or basal ganglia) NEAR/5 (isch?emi* or infarct*)):ti,ab,kw
15 (((lacunar or cortical) NEAR/5 infarct*)):ti,ab,kw
16 ((intracran* or intracerebral) NEAR/3 (thrombo* or thrombus* or embol*)):ti,ab,kw
17 SAH:ti,ab,kw
18 ((brain* or cerebr* or cerebell* or intracerebral or intracran* or parenchymal or intraventricular or infratentorial or supratentorial or basal gangli* or subarachnoid or putaminal or putamen or posterior fossa) NEAR/5 (haemorrhage* or hemorrhage* or bleed* or rupture* NEAR/3 aneurysm)):ti,ab,kw
19 (tia*1 or transi* NEAR/3 (isch?emia* attack or brain isch?emia* or cerebral isch?emia* or CVA* or cerebral vasc* or cerebrovasc*)):ti,ab,kw
20 #13 or #14 or #15 or #16 or #17 or #18 or #19
21 #12 and #20

SCOPUS (www.scopus.com) search strategy [inception to 9th February 2017]

(((TITLE-ABS-KEY ("cold sore*")) OR (TITLE-ABS-KEY ("genit* herpes*" OR "genit* sores")) OR (TITLE-ABS-KEY (varicella OR chickenpox OR "chicken pox" OR shingles OR vzv OR zoster)) OR (TITLE-ABS-KEY (cmv OR cytomegalovirus)) OR (TITLE-ABS-KEY ("B lymphotropic virus*" OR roseola OR "sixth disease" OR "exanthema subitum" OR "exanthem criticum" OR roseolovirus OR pseudorubella OR "three?day fever")) OR (TITLE-ABS-KEY (ebv OR epstein-barr OR "burkitt W/5 lymphoma*" OR "glandular fever" OR "infectious mono*" OR mononucleosis OR "hair* leukoplak*" OR ohl)) OR (TITLE-ABS-KEY ("kaposi* sarcoma*" OR "Primary effusion W/2 lymphoma*" OR "body cavity W/2 lymphoma*")) OR (TITLE-ABS-KEY ((hhv W/1 ("1" OR "2" OR "3" OR "4" OR "5" OR "6" OR "7" OR "8")) OR (hhv?1 OR hhv?2 OR hhv?3 OR hhv?4 OR hhv?5 OR hhv?6 OR hhv?7 OR hhv?8)))) OR (TITLE-ABS-KEY (hsv?1 OR "HSV 1" OR hsv?2 OR "HSV 2")) OR (TITLE-ABS-KEY (herpes*)) OR (TITLE-ABS-KEY (ac?clovir OR zovirax OR valac?clovir OR valtrex OR famc?clovir OR famvir OR penc?clovir OR ganc?clovir OR cidofovir OR foscarnet* OR valganc?clovir OR lubocavir OR brivudin OR docosanol OR sorivudine OR idoxuridine OR trifluridine))) AND ((TITLE-ABS-KEY (stroke OR cva OR (cerebrovasc* AND (disease OR event OR accident OR attack OR injury)))) OR (TITLE-ABS-KEY ((brain* OR cerebr* OR cerebell* OR cortical OR vertebrobasilar OR hemispher* OR intracran* OR intracerebral OR infratentorial OR supratentorial OR mca OR "anterior circulation" OR "posterior circulation" OR "basal ganglia") W/5 (isch?emi* OR infarct*))) OR (TITLE-ABS-KEY (((lacunar OR cortical) W/5 infarct*))) OR (TITLE-ABS-KEY ((intracran* OR intracerebral) W/3 (thrombo* OR thrombus* OR embol*))) OR (TITLE-ABS-KEY (sah)) OR (TITLE-ABS-KEY ((brain* OR cerebr* OR cerebell* OR intracerebral OR intracran* OR parenchymal OR intraventricular OR infratentorial OR supratentorial OR "basal gangli*" OR subarachnoid OR putaminal OR putamen OR "posterior fossa") W/5 (haemorrhage* OR hemorrhage* OR bleed* OR "rupture* aneurysm"))) OR (TITLE-ABS-KEY (tia*1 OR transi* W/3 ("isch?emia* attack" OR "brain isch?emia*" OR "cerebral isch?emia*" OR cva* OR "cerebral vasc*" OR cerebrovasc*))))

Web of Science [inception to 8th February 2017]

1 TS=((stroke or cva or (cerebrovasc* AND (disease or event or accident or attack or injury)))) OR TS=(((brain* or cerebr* or cerebell* or cortical or vertebrobasilar or hemispher* or intracran* or intracerebral or infratentorial or supratentorial or MCA or "anterior circulation" or "posterior circulation" or "basal ganglia") NEAR/5 (isch?emi* or infarct*))) OR TS=((((lacunar or cortical) NEAR/5 infarct*))) OR TS=(((intracran* or intracerebral) NEAR/3 (thrombo* or thrombus* or embol*))) OR TS=((SAH)) OR TS=(((brain* or cerebr* or cerebell* or intracerebral or intracran* or parenchymal or intraventricular or infratentorial or supratentorial or "basal gangli*" or subarachnoid or putaminal or putamen or "posterior fossa") NEAR/5 (haemorrhage* or hemorrhage* or bleed* or rupture* NEAR/3 aneurysm))) OR TS=((stroke or cva or (cerebrovasc* AND (disease or event or accident or attack or injury)))) OR TS=((tia*1 or transi* NEAR/3 ("isch?emia* attack" or "brain isch?emia*" or "cerebral isch?emia*" or CVA* or "cerebral vasc*" or cerebrovasc*)))

2 TS=(("cold sore*")) OR TS=(("genit* herpes*" or "genit* sores")) OR TS=((varicella or chickenpox or "chicken pox" or shingles or VZV or zoster)) OR TS=((CMV or cytomegalovirus)) OR TS=(("B lymphotropic virus*" or roseola or "sixth disease" or "exanthema subitum" or "exanthem criticum" or Roseolovirus or pseudorubella or "three?day fever")) OR TS=((EBV or epstein-barr or burkitt NEAR/5 lymphoma* or "glandular fever" or "infectious mono*" or mononucleosis or "hair* leukoplak*" or OHL)) OR TS=(("kaposi* sarcoma*" or "Primary effusion" NEAR/2 lymphoma* or "body cavity" NEAR/2 lymphoma*)) OR TS=(((HHV NEAR/1 ("1" or "2" or "3" or "4" or "5" or "6" or "7" or "8")) or (HHV?1 or HHV?2 or HHV?3 or HHV?4 or HHV?5 or HHV?6 or HHV?7 or HHV?8))) OR TS=((herpes*)) OR TS=((ac?clovir or Zovirax or valac?clovir or valtrex or famc?clovir or famvir or penc?clovir or ganc?clovir or cidofovir or foscarnet* or valganc?clovir or lubocavir or brivudin or Docosanol or Sorivudine or Idoxuridine or Trifluridine))

3 #1 and #2
