# Supplementary material for: Association of herpesviruses and stroke: Systematic review and meta-analysis
Source: PLoS One. 2018 Nov 21;13(11):e0206163. doi: 10.1371/journal.pone.0206163 (PMC6248930; doi:10.1371/journal.pone.0206163)
Supplement: S2 Appendix — (DOCX) [file pone.0206163.s002.docx]

## **S2 Appendix: Changes to the original protocol**

## We added a relevant bias domains to our risk of bias approach: Reverse causation was an additional domain not included in the protocol.

In the original protocol we said each domain would be classified as either ‘high risk’ (if criterion are very inadequately addressed), ‘low risk’ (if criterion are adequately addressed) or ‘unclear risk’. We have now added an additional criterion - ‘moderate risk’ (if criterion are somewhat inadequately addressed).

1. Once the risk of bias assessment was completed, a summary risk of bias table was produced; where a domain had more than one item for assessment (e.g. the second domain, “selection of participants”, had two items - participation bias and selection of controls) the highest risk of bias judgment was used in the summary table.
2. In the original protocol, we had written, “Studies were included if the primary outcome was stroke (first or subsequent).” However, if a study met all other criteria but stroke was only a secondary outcome, we decided it should be included. Therefore, we have revised it to, “Studies were included if stroke (first ever or subsequent) was an outcome”.
