# Supplementary material for: Association of herpesviruses and stroke: Systematic review and meta-analysis
Source: PLoS One. 2018 Nov 21;13(11):e0206163. doi: 10.1371/journal.pone.0206163 (PMC6248930; doi:10.1371/journal.pone.0206163)
Supplement: S3 Appendix — (DOCX) [file pone.0206163.s003.docx]

## S3: Appendix

## **Data items on the following five domains were extracted**

1. *Population:* characteristics of the study population (e.g. mean/median age, ethnic distribution, immune status), inclusion and exclusion criteria;
2. *Exposure:* definition and identification of human herpesvirus exposure, number of exposed subjects;
3. *Comparators:* definition and identification of unexposed individuals, number of unexposed subjects;
4. *Outcomes:* definition and identification of primary (stroke) and secondary outcomes (stroke subtypes or TIA), number of subjects with outcome;
5. *Study characteristics:* authors, publication year, setting/source of participants, design, methods of recruitment and sampling, period of study, length of follow-up time (if relevant), aims and objectives.
