# Supplementary material for: Association of herpesviruses and stroke: Systematic review and meta-analysis
Source: PLoS One. 2018 Nov 21;13(11):e0206163. doi: 10.1371/journal.pone.0206163 (PMC6248930; doi:10.1371/journal.pone.0206163)
Supplement: S4 Appendix — (DOCX) [file pone.0206163.s004.docx]

S4 Appendix

**Grade assessment of quality: down/ up-grading reasons**

1. Risk of bias:
   - Not serious if more than half the studies have no domain at high risk of bias.
   - Very serious if studies with two or more domains at high risk of bias represent more than 50% of the total studies and contribute more than 50% to any meta-analyses.
   - Serious if studies fall between not serious and very serious.
2. Inconsistency not serious if have 0, serious if have 1, and very serious if have two or more of the following:
   - Heterogeneity is moderate (I² ~30-60%), substantial (50-90%) or considerable (75-100%).
   - Wide variance of point estimates across studies.
   - Minimal/no overlap of the confidence intervals.
3. Indirectness: Direct evidence consists of research that directly compares the interventions which we are interested in, delivered to the populations in which we are interested, and measures the outcomes important to patients. The effect on overall quality of evidence will vary depending on how indirect the evidence is. Rough guide - not serious if have 0, serious if have 1, and very serious if have two of the following:
   - An indirect comparison (for example study A compares to a placebo and study B compares to a different drug).
   - Studies differ in terms of population (e.g. be restricted to immunosuppressed patients).
   - Studies differ in terms of exposure definition (e.g. for example use different methods to ascertain herpesvirus exposures).
   - Studies differ in terms of outcome measures (e.g. for example be restricted to certain time-frames or have a different definition of stroke).
4. Imprecision: where studies have low power and consequently wide confidence intervals (based on guidelines from the Cochrane Back Group).
   - Serious imprecision: Wide confidence intervals
   - Very serious imprecision: Very wide confidence intervals

Upgrading reasons include

1. Large effect:
   - None: most effect estimates are less than two.
   - Strong association: ES* >2 or <0.5 (based on direct evidence, with no plausible confounders)
   - Very strong association: ES* >4 or <0.2 (based on direct evidence with no serious problems with risk of bias or precision, i.e. with (sufficiently narrow confidence intervals)
2. Plausible confounding
   - Would reduce demonstrated effect: e.g. If, for instance, only sicker patients receive an intervention or exposure, yet they still fare better, it is likely that the actual intervention or exposure effect is even larger than the data suggest (confounding by indication) Patients given antivirals are likely to be less healthy so this could reduce the demonstrated effect seen in patients given antivirals.
   - Would suggest spurious effect: When confounding is expected to increase the effect but no effect was observed.
3. Dose response gradient (yes/no)
