# Supplementary material for: Association of herpesviruses and stroke: Systematic review and meta-analysis
Source: PLoS One. 2018 Nov 21;13(11):e0206163. doi: 10.1371/journal.pone.0206163 (PMC6248930; doi:10.1371/journal.pone.0206163)
Supplement: S5 Appendix — (DOCX) [file pone.0206163.s005.docx]

S5 Appendix: References of selected studies

^1-41^

1. Breuer J, Pacou M, Gautier A, Brown MM. Herpes zoster as a risk factor for stroke and TIA: a retrospective cohort study in the UK. Neurology. 2014 Jul 08;83(2):e27-33.

2. Calabrese LH, Xie F, Yun H, et al. Herpes Zoster and the Risk of Stroke in Patients With Autoimmune Diseases. Arthritis Rheumatol. 2017 Feb;69(2):439-46.

3. Kang JH, Ho JD, Chen YH, Lin HC. Increased Risk of Stroke After a Herpes Zoster Attack A Population-Based Follow-Up Study. Stroke. 2009 Nov;40(11):3443-8.

4. Kwon SU, Yun SC, Kim MC, et al. Risk of stroke and transient ischaemic attack after herpes zoster. Clin Microbiol Infect. 2016 Jun;22(6):542-8.

5. Lin HC, Chien CW, Ho JD. Herpes zoster ophthalmicus and the risk of stroke A population-based follow-up study. Neurology. 2010 Mar 9;74(10):792-7.

6. Sreenivasan N, Basit S, Wohlfahrt J, et al. The Short- and Long-Term Risk of Stroke after Herpes Zoster - A Nationwide Population-Based Cohort Study. PloS one. 2013 Jul 17;8(7).

7. Sundstrom K, Weibull CE, Soderberg-Lofdal K, Bergstrom T, Sparen P, Arnheim-Dahlstrom L. Incidence of herpes zoster and associated events including stroke-a population-based cohort study. Bmc Infectious Diseases. 2015 Oct 31;15.

8. Tseng HF, Smith N, Jacobsen S. Incidence of Stroke Following Herpes Zoster among Population 50 Years and Older. J Epidemiol Commun H. 2011 Aug;65:A167-A.

9. Yawn BP, Wollan PC, Nagel MA, Gilden D. Risk of Stroke and Myocardial Infarction After Herpes Zoster in Older Adults in a US Community Population. Mayo Clin Proc. 2016 Jan;91(1):33-44.

10. Yen YF, Chen M, Jen I, et al. Association of HIV and Opportunistic Infections With Incident Stroke: A Nationwide Population-Based Cohort Study in Taiwan. J Acquir Immune Defic Syndr. 2017 Feb 01;74(2):117-25.

11. Langan S, Minassian C, Smeeth L, Thomas S. Risk of stroke following herpes zoster: a self-controlled case-series study. J Invest Dermatol. 2014 Sep;134:S39-S.

12. Minassian C, Thomas SL, Smeeth L, Douglas I, Brauer R, Langan SM. Acute Cardiovascular Events after Herpes Zoster: A Self-Controlled Case Series Analysis in Vaccinated and Unvaccinated Older Residents of the United States. PLoS medicine. 2015 Dec;12(12).

13. Schink T, Behr S, Thone K, Bricout H, Garbe E. Risk of Stroke after Herpes Zoster - Evidence from a German Self-Controlled Case-Series Study. PloS one. 2016;11(11):e0166554.

14. Coles KA, Knuiman MW, Plant AJ, Riley TV, Smith DW, Divitini ML. A prospective study of infection and cardiovascular diseases: the Busselton Health Study. Eur J Cardiovasc Prev Rehabil. 2003 Aug;10(4):278-82.

15. Fagerberg B, Gnarpe J, Gnarpe H, Agewall S, Wikstrand J. Chlamydia pneumoniae but not cytomegalovirus antibodies are associated with future risk of stroke and cardiovascular disease: a prospective study in middle-aged to elderly men with treated hypertension. Stroke. 1999 Feb;30(2):299-305.

16. Gonzalez-Quijada S, Salazar-Thieroldt E, Mora-Simon MJ. Persistent Q fever and ischaemic stroke in elderly patients. Clin Microbiol Infect. 2015 Apr;21(4):362-7.

17. Huang ZR, Yu LP, Yang XC, et al. Human cytomegalovirus linked to stroke in a Chinese population. CNS Neurosci Ther. 2012 Jun;18(6):457-60.

18. Ķēniņa V, Auce P, Priede Z, et al. Cytomegalovirus chronic infection as a risk factor for stroke: a prospective study. Proceedings of the Latvian Academy of Sciences Section B Natural, Exact, and Applied Sciences2010. p. 133.

19. Oliveras A, Roquer J, Puig JM, et al. Stroke in renal transplant recipients: epidemiology, predictive risk factors and outcome. Clin Transplant. 2003 Feb;17(1):1-8.

20. Shen X, Zhang W, Zhang S, Cui M. The detection and clinical significance of serum HCMV IgM in patients with atherosclerosis and cerebral infarction. Journal of China Medical University. 2011;40(4):346-8.

21. Smieja M, Gnarpe J, Lonn E, et al. Multiple infections and subsequent cardiovascular events in the Heart Outcomes Prevention Evaluation (HOPE) Study. Circulation. 2003 Jan 21;107(2):251-7.

22. Tarnacka B, Gromadzka G, Czlonkowska A. Increased circulating immune complexes in acute stroke: the triggering role of Chlamydia pneumoniae and cytomegalovirus. Stroke. 2002 Apr;33(4):936-40.

23. Yi L, Wang DX, Feng ZJ. Detection of human cytomegalovirus in atherosclerotic carotid arteries in humans. J Formos Med Assoc. 2008 Oct;107(10):774-81.

24. Zheng L, Sun Z, Zhang X, et al. Human cytomegalovirus increases the risk of future hemorrhagic but not ischemic stroke - A nested case-control study. Circulation Journal. 2016;80(10):2235-9.

25. Ziemann M, Heringlake M, Lenor P, et al. Cytomegalovirus Serostatus as Predictor for Adverse Events After Cardiac Surgery: A Prospective Observational Study. Journal of Cardiothoracic and Vascular Anesthesia. 2016.

26. Yen Y, Jen I, Chen M, et al. Association of cytomegalovirus end-organ disease with stroke in people living with HIV/AIDS: a nationwide population-based cohort study. PloS one. 2016;11(3).

27. Al-Ghamdi A. Role of herpes simplex virus-1, cytomegalovirus and Epstein-Barr virus in atherosclerosis. Pak. 2012 Jan;25(1):89-97.

28. Elkind MS, Hills NK, Glaser CA, et al. Herpesvirus Infections and Childhood Arterial Ischemic Stroke: Results of the VIPS Study. Circulation. 2016 Feb 23;133(8):732-41.

29. Elkind MS, Ramakrishnan P, Moon YP, et al. Infectious burden and risk of stroke: the northern Manhattan study. Archives of Neurology. 2010 Jan;67(1):33-8.

30. Kis Z, Sas K, Gyulai Z, et al. Chronic infections and genetic factors in the development of ischemic stroke. New Microbiol. 2007 Jul;30(3):213-20.

31. Li ML, Li ZZ, Li SP, He JY, Wang YX. Stroke in progression, infection of herpes virus and neurologic impairment inhibited with antivirus druggery. Chinese Journal of Clinical Rehabilitation. 2005 07 Apr;9(13):158-61.

32. Ozturk A, Gunes M, Altinoz Aytar A, Ozturk CE, Ankarali H. Are some chronic infections probable risk factors for acute ischemic stroke? Turkiye Klinikleri Journal of Medical Sciences. 2013;33(3):726-31.

33. Ridker PM, Hennekens CH, Stampfer MJ, Wang F. Prospective study of herpes simplex virus, cytomegalovirus, and the risk of future myocardial infarction and stroke. Circulation. 1998 Dec 22-29;98(25):2796-9.

34. Sealy-Jefferson S, Gillespie BW, Aiello AE, Haan MN, Morgenstern LB, Lisabeth LD. Antibody Levels to Persistent Pathogens and Incident Stroke in Mexican Americans. PloS one. 2013 14 Jun;8 (6) (no pagination)(e65959).

35. Asiki G, Stockdale L, Kasamba I, et al. Pilot study of antibodies against varicella zoster virus and human immunodeficiency virus in relation to the risk of developing stroke, nested within a rural cohort in Uganda. Trop Med Int Health. 2015 Oct;20(10):1306-10.

36. Askalan R, Laughlin S, Mayank S, et al. Chickenpox and stroke in childhood: a study of frequency and causation. Stroke. 2001 Jun;32(6):1257-62.

37. Sebire G, Meyer L, Chabrier S. Varicella as a risk factor for cerebral infarction in childhood: a case-control study. Ann Neurol. 1999 May;45(5):679-80.

38. Thomas SL, Minassian C, Ganesan V, Langan SM, Smeeth L. Chickenpox and risk of stroke: a self-controlled case series analysis. Clinical Infectious Diseases. 2014 Jan;58(1):61-8.

39. Baxter R, Tran TN, Hansen J, et al. Safety of ZostavaxTM-A cohort study in a managed care organization. Vaccine. 2012 19 Oct;30(47):6636-41.

40. Tseng HF, Liu A, Sy L, et al. Safety of zoster vaccine in adults from a large managed-care cohort: a Vaccine Safety Datalink study. J Intern Med. 2012 May;271(5):510-20.

41. Donahue JG, Kieke BA, Yih WK, et al. Varicella vaccination and ischemic stroke in children: is there an association? Pediatrics. 2009 Feb;123(2):e228-34.
