# Supplementary material for: Association of herpesviruses and stroke: Systematic review and meta-analysis
Source: PLoS One. 2018 Nov 21;13(11):e0206163. doi: 10.1371/journal.pone.0206163 (PMC6248930; doi:10.1371/journal.pone.0206163)

S1 Fig: Effect of clinically diagnosed ophthalmic zoster on stroke risk, by study design and length of follow-up.
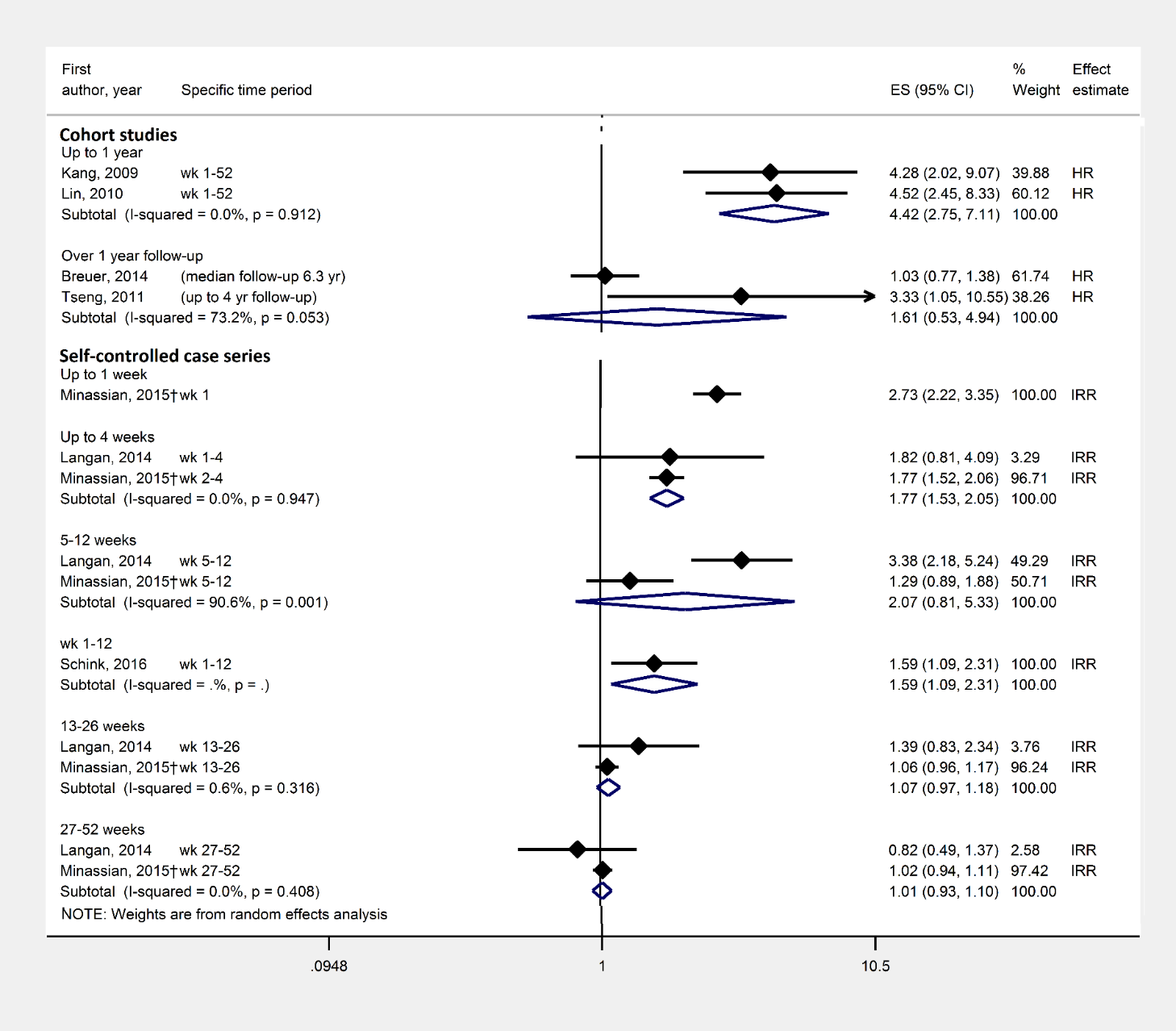

Supplement: S1 Fig — (DOCX) [file pone.0206163.s006.docx]
