# Supplementary material for: Association of herpesviruses and stroke: Systematic review and meta-analysis
Source: PLoS One. 2018 Nov 21;13(11):e0206163. doi: 10.1371/journal.pone.0206163 (PMC6248930; doi:10.1371/journal.pone.0206163)
Supplement: S3 Fig — (DOCX) [file pone.0206163.s008.docx]

S3 Fig: Effect of zoster on stroke risk by length of follow-up and age group (all studies are cohort design)


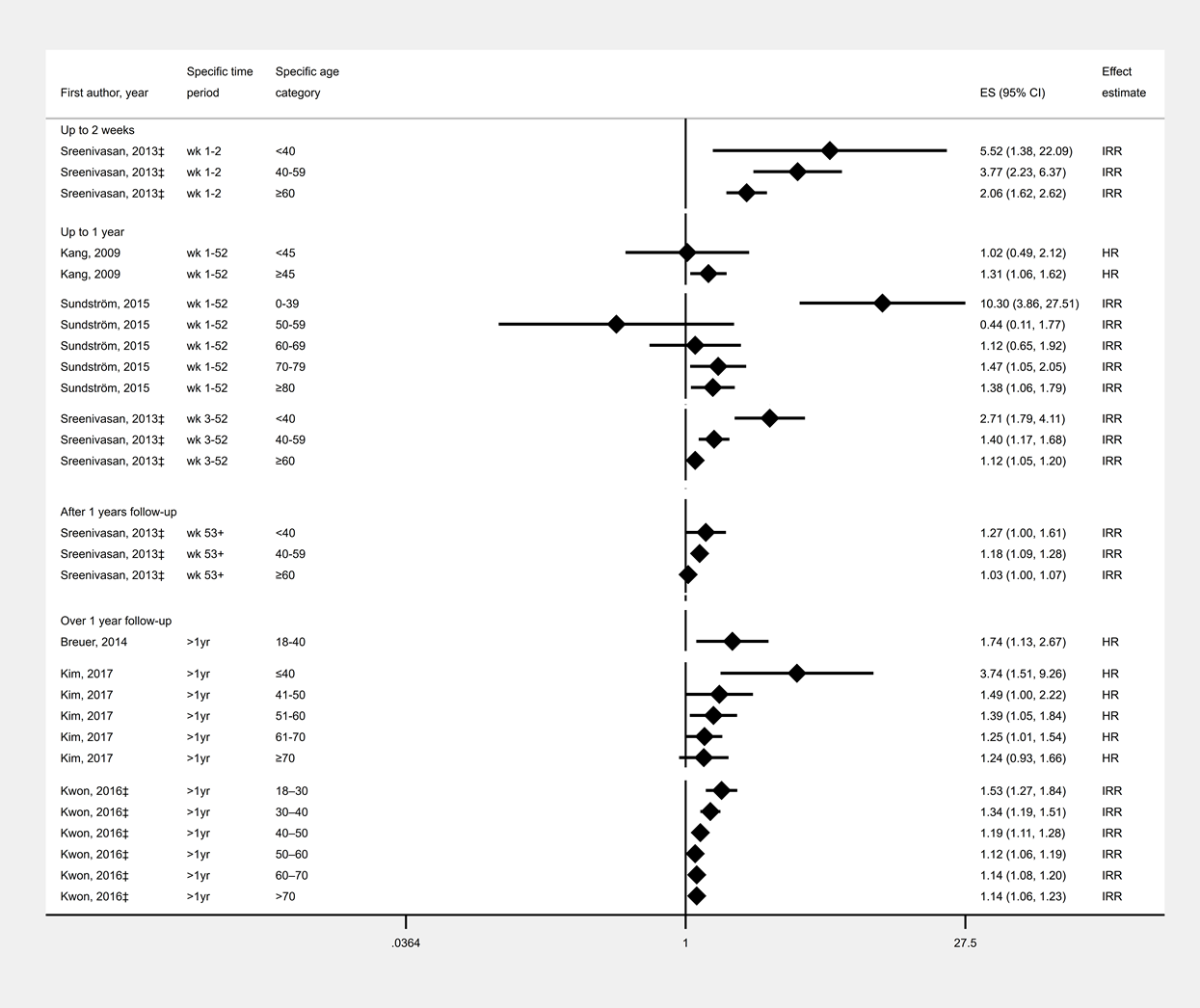


= Age less than 40 years = Age over 40 years

†Outcome was ischaemic stroke ‡Outcome was stroke/TIA

•Study population was immunosuppressed

‼No age adjustment/matching for age

*Comparator group was pooled rate in year 2-6 following HZ **Comparator group was person time 366-730days after HZ
