# Supplementary material for: Association of herpesviruses and stroke: Systematic review and meta-analysis
Source: PLoS One. 2018 Nov 21;13(11):e0206163. doi: 10.1371/journal.pone.0206163 (PMC6248930; doi:10.1371/journal.pone.0206163)
Supplement: S4 Fig — (DOCX) [file pone.0206163.s009.docx]

S4 Fig: Effect of zoster on stroke risk by length of follow-up and gender (all studies are cohort design)

= Females = Males

†Outcome was ischaemic stroke ‡Outcome was stroke/TIA
