# Supplementary material for: Association of herpesviruses and stroke: Systematic review and meta-analysis
Source: PLoS One. 2018 Nov 21;13(11):e0206163. doi: 10.1371/journal.pone.0206163 (PMC6248930; doi:10.1371/journal.pone.0206163)
Supplement: S6 Fig — (DOCX) [file pone.0206163.s011.docx]

**S6 Fig: Assessment of publication bias for CMV IgG seropositivity as a risk factor for stroke.** Funnel plot of the log odds ratio plotted against the standard error of the log odds ratio for 10 studies reporting the effect of CMV IgG seropositivity on stroke risk (dotted line represents pseudo 95% confidence limits).

Note: raw event data not available for 1 study (Elkind, 2010), therefore cannot conduct statistical test using Peters test. Using Begg's test for small-study effects: P=0.180.
