# Supplementary material for: Association of herpesviruses and stroke: Systematic review and meta-analysis
Source: PLoS One. 2018 Nov 21;13(11):e0206163. doi: 10.1371/journal.pone.0206163 (PMC6248930; doi:10.1371/journal.pone.0206163)
Supplement: S1 Table — (DOCX) [file pone.0206163.s012.docx]

| S1 Table: Exploring statistical heterogeneity identified in meta-analyses | | | | | |
| --- | --- | --- | --- | --- | --- |
|  | **No. of studies** | **Summary RR (95% CI)** | | **I²** | **P-value from meta-regression** |
|  | | | | |  |
| **Herpes zoster – Cohort studies with over 1 year follow-up** | | | | |  |
| Results from primary analysis including all studies | 7 | 1.11 (1.02-1.22) | 72.5% | | - |
| Remove studies at high risk of bias (Kwon, 2016; Kim, 2017; Patterson, 2018)) | 4 | 1.02 (0.98-1.07) | <0.01% | | - |
|  |  |  |  | |  |
| **Herpes zoster – SCCS with 5-12 weeks follow-up** |  |  |  | |  |
| Results from primary analysis including all studies | 3 | 1.24 (1.12-1.38) | 62.2% | | - |
| *No studies at high risk of bias* | - | - | - | | - |
|  |  |  |  | |  |
| **Herpes zoster – SCCS with 13-26 weeks follow-up** |  |  |  | |  |
| Results from primary analysis including all studies | 3 | 1.09 (0.99-1.21) | 67.1% | | - |
| *No studies at high risk of bias* | - | - | - | | - |
|  |  |  |  | |  |
| **CMV – IgG seropositivity in case control studies** |  |  |  | |  |
| Results from primary analysis including all studies | 6 | 1.40 (0.67-2.96) | 78.8% | | - |
| *All studies at high risk of bias* | - | - | - | | - |
|  |  |  |  | |  |
| **CMV - DNA** |  |  |  | |  |
| Results from primary analysis including all studies | 3 | 2.34 (0.95-5.74) | 81.4% | | - |
| *2 studies at high risk of bias: cannot analyse single remaining study* | - | - | - | | - |
|  |  |  |  | |  |

Note: There were too few studies using PCR to compare studies using PCR versus ELISA techniques to identify CMV infection. If a meta-analysis combined only two studies we did not explore heterogeneity
