# Supplementary material for: Association of herpesviruses and stroke: Systematic review and meta-analysis
Source: PLoS One. 2018 Nov 21;13(11):e0206163. doi: 10.1371/journal.pone.0206163 (PMC6248930; doi:10.1371/journal.pone.0206163)
Supplement: S2 Table — (PDF) [file pone.0206163.s013.pdf]

Table 2: Risk of bias summary with judgement and short justification about each risk of bias domain (for the appendices)

	Confounding	Selection of participants		Misclassification of variables										Bias due to missing data		Reverse causation	Generalisability	Study Power
				Exposure				Outcome				Covariates						
				Differential			Non-differential	Differential			Non-differential	Differential	Non-differential					
	Age and other confounders	Participation bias	Selection of controls	Recall bias	Observer bias	Ascertainment bias		Recall bias	Observer bias	Ascertainment bias				Differential loss to follow up	Exclusion of individuals with missing data			
Criteria for risk of bias assessment	**Low:** Adjusted for age with several categories or continuous if appropriate. Measured at baseline and time-updated if necessary. **Moderate:** Age-adjusted but with less detail (eg binary). **High:** Not adjusted for age.	[illegible] participation (e.g. medical record review), or (2) random sample. **Moderate:** Non-random sample, with evidence that those included and not included in the study have different characteristics in terms of age and gender. **High:** Non-random sample, with no comparison of [illegible]	**Low:** controls selected using random sampling from the population from which the cases arose. **Moderate:** Controls not selected from the population from which the cases arose OR controls not selected using random sampling. **High:** probability of selection as a control likely to be affected by exposure status (known or unknown).	**Low:** exposure status defined before occurrence of stroke. **High:** exposure status defined by patient recall of exposure in context of recent stroke	**Low:** exposure status defined before occurrence of stroke OR exposure status defined by observer blinded to outcome (stroke) status or exposure defined from test result **High:** exposure status defined by observer unblinded to outcome (stroke) status, without clear objective criteria to apply.	**Low:** exposure status defined before occurrence of stroke. **High:** participants with stroke are more or less likely to be tested for exposures.	**Low:** All members of study assessed for exposure at baseline. Exposure defined using laboratory criteria. **High**: allocation of exposure status relies on exposure having been diagnosed as part of routine medical care: exposure may therefore be missed - important for varicella.	**Low:** stroke status defined prior to exposure status assigned. **High**: stroke status defined by patient recall of stroke in context of herpes virus exposure.	**Low:** stroke status defined prior to exposure status assigned. **High:** stroke status defined by observer in context likely to be influenced by herpesvirus status.	**Low:** ascertainment of stroke unlikely to be influenced by herpesvirus status. **High**: ascertainment of stroke likely to be influenced by herpesvirus status (possibly via health-seeking behaviour).	**Low:** Active data collection of outcome, or outcome unlikely to be missed or presents validation results of >70% sensitivity and specificity. **High:** Unclear or unvalidated method of diagnosis.	Could the misclassification vary by case /control or exposed/ unexposed status?		[illegible] follow up, or (2) ≥80% follow up, or (3) 70-80% follow up with a comparison (min age, sex) showing similar characteristics between those included and not included in the study. Moderate: 70-80% follow up with a comparison (min age, sex) showing dissimilar characteristics between those included and not included. High: <70% follow-up that will likely impact [illegible]	**Low:** None or very low percentage of missing data, or appropriate missing data technique used). Sensitivity analysis performed to assess potential impact of missingness. **Moderate**: Substantial missing data (15-25%) with no or inappropriate method used. **High:** Large amount of missing data (>25 %) with no discussion /attempt to assess impact, or [illegible]	**Low:** exposure defined prior to ascertainment of stroke. **High**: exposure defined after the stroke defined as the study outcome.		**Low:** Power calculation included or large numbers with narrow confidence intervals for main effect. **High:** No power calculation or small study with small numbers and few outcomes for main effect.
Varicella zoster virus reactivation- zoster																		
Breuer, 2014	Low: Matched on age within 2 years, adjusted for various risk factors.	Low: automated participation, comparison cohort selected from a random sample of unexposed patients	N/A	Low: Exposure ascertained from pre-existing medical records - doesn't rely on patient recall in context of stroke	Low: used pre-specified codes to define HZ exposure status before occurrence of stroke	Low: exposure diagnosis pre-dated stroke	Low: Medical diagnosis of HZ in medical records, with care taken to reduce misclassification of herpes simplex as HZ.	Low: stroke identified from medical records - doesn't rely on patient recall	Low: clinical diagnosis of stroke unlikely to be severely affected by recent HZ reactivation.	Low: exposure status (HZ reactivation) unlikely to affect primary care attendance with stroke	Low: Medical diagnosis of stroke. Stroke type (ischaemic/haemorrhagic) missing 80% of sample. Hospitalisation data not available.	Low: Capture unlikely to differ by HZ exposure status.	Low: ascertained from GP records.	Low: automated follow-up	Low: BMI, cholesterol level, and smoking status contained missing data, however not major confounders. Location of HZ rarely reported - reduced value of HZ location and stroke association.	Low: exposure defined prior to development of stroke.	Applicable to adults	Low: large study, well powered.
Calabrese, 2017	Moderate: categorisation of age not described. Range of confounders considered	Low: automated participation, comparison cohort selected from a random sample of unexposed patients	N/A	Low: Exposure ascertained from pre-existing medical records - doesn't rely on patient recall in context of stroke	Low: used pre-specified codes to define HZ exposure status before occurrence of stroke	Low: exposure diagnosis pre-dated stroke	Low: Medical diagnosis of HZ in medical records: PPV reported at >85%.	Low: stroke identified from medical records - doesn't rely on patient recall	Low: clinical diagnosis of stroke unlikely to be severely affected by recent HZ reactivation.	Low: exposure status (HZ reactivation) unlikely to affect primary care attendance with stroke	Low: Medical diagnosis of stroke. Stroke type (ischaemic/haemorrhagic) available <50% of sample.	Unclear: ascertainment of covariates not described in detail.	Unclear: ascertainment of covariates not described in detail.	Low: automated follow-up	Unclear: missing data not described	Low: exposure defined prior to development of stroke.	Applicable to patients with specific autoimmune conditions	Low: Large study with narrow confidence intervals
Hosamirudsari, 2018	Moderate: categorisation of age not described. Range of confounders considered	High: consecutive sampling and no information on eligible patients not included in the analyses (eg refusing to participate)	Moderate: controls selected from the population giving rise to the cases (same hospital), however not clear whether controls were recruited randomly	High: exposure status defined by patient recall of exposure in context of recent stroke	High: exposure status defined by observer unblinded to outcome (stroke) status, without clear objective criteria to apply.	Low: exposure ascertained for cases and controls	High: misclassification of HZ possible as relies on self-report	Low: stroke identified from diagnostic imaging - doesn't rely on patient recall.	Low: clinical diagnosis of stroke unlikely to be severely affected by recent HZ reactivation.	Low: exposure status (HZ reactivation) unlikely to affect attendance with stroke	Low: Medical diagnosis of stroke.	Unclear: ascertainment of covariates not described in detail.	Unclear: ascertainment of covariates not described in detail.	Low: case-control study, therefore no follow-up.	Unclear: missing data not described	Low: exposure defined in previous 6 months	Applicable to adults	High: small study, power calculation not described, wide confidence intervals.
Kang, 2009	Low: Matched exposed and unexposed on age (as a continuous variable).	Low: automated participation from a sample of large database: sample is similar in terms of age, sex and healthcare costs to all enrolees.	N/A	Low: Exposure ascertained from pre-existing medical records - doesn't rely on patient recall in context of stroke	Low: used pre-specified codes to define HZ status, ascertained prior to stroke developed	Low: exposure status defined before occurrence of stroke.	Low: misclassification of HZ possible (eg with herpes simplex) however assumed to be minimal.	Low: stroke identified from medical records - doesn't rely on patient recall	Low: clinical diagnosis of stroke unlikely to be severely affected by recent HZ reactivation.	Low: exposure status unlikely to affect primary care attendance with stroke	Low: Medical diagnosis of stroke.	Low: Capture unlikely to differ by HZ exposure status.	Low: good ascertainment of covariates	Low: automated follow-up	Unclear: missing data not described	Low: exposure defined prior to development of stroke.	Applicable to Taiwanese adults	Low: large study, well powered.
Kim, 2017	High: age adjustment unclear - yet did adjust for other potential confounders.	Low: automated participation using random sample	N/A	Low: Exposure ascertained from pre-existing medical records - doesn't rely on patient recall in context of stroke	Low: exposure status defined before occurrence of stroke	Low: exposure diagnosis pre-dated stroke	Low: Medical diagnosis of HZ in medical records. However time unexposed may be misclassified as exposed, due to the study design.	Low: stroke identified from medical records - doesn't rely on patient recall	Low: clinical diagnosis of stroke unlikely to be severely affected by recent HZ reactivation.	Low: exposure status (HZ reactivation) unlikely to affect primary care attendance with stroke	Low: Medical diagnosis of stroke.	Low: Adjusted for age and vascular risk factors. Capture unlikely to vary by HZ status	Unclear: ascertainment of covariates not described in detail.	Low: automated follow-up	Unclear: missing data not described	Low: exposure defined prior to development of stroke.	Unknown - age not described	Low: Large study with narrow confidence intervals
Kwon, 2016	High: age adjustment unclear - yet did adjust for other potential confounders.	Low: automated participation using random sample	N/A	Low: Exposure ascertained from pre-existing medical records - doesn't rely on patient recall in context of stroke	Low: exposure status defined before occurrence of stroke	Low: exposure diagnosis pre-dated stroke	Low: Medical diagnosis of HZ in medical records. However time unexposed may be misclassified as exposed, due to the study design.	Low: stroke identified from medical records - doesn't rely on patient recall	Low: clinical diagnosis of stroke unlikely to be severely affected by recent HZ reactivation.	Low: exposure status (HZ reactivation) unlikely to affect primary care attendance with stroke	Low: Medical diagnosis of stroke. However, stroke type(ischaemic/ haemorrhagic) available 60% of sample.	Low: Adjusted for age and vascular risk factors. Capture unlikely to vary by HZ status	Unclear: ascertainment of covariates not described in detail.	Low: automated follow-up	Unclear: missing data not described	Low: exposure defined prior to development of stroke.	Applicable to adults	Low: Large study with narrow confidence intervals
Langan, 2014	Low: Adjusted for age in 5 year bands. Study design controls for time-independent confounding.	Low: automated participation	N/A	Low: Exposure ascertained from pre-existing medical records - doesn't rely on patient recall in context of stroke	Low: exposure status defined before occurrence of stroke	Low: exposure diagnosis pre-dated stroke	Low: Medical diagnosis of HZ in medical records. But misclassification of HZO is likely.	Low: stroke identified from medical records - doesn't rely on patient recall	Low: clinical diagnosis of stroke unlikely to be severely affected by recent HZ reactivation.	Low: exposure status (HZ reactivation) unlikely to affect primary care attendance with stroke	Low: Medical diagnosis of stroke with thorough exclusions to avoid misclassification.	Low: Capture unlikely to differ by HZ exposure status.	Low: adjusted for age only	Low: automated follow-up	Low: no missing data.	Low: exposure defined prior to development of stroke.	Applicable to adults	Low: Large study with narrow confidence intervals

	Confounding	Selection of participants		Misclassification of variables										Bias due to missing data		Reverse causation	Generalisability	Study Power
	Age and other confounders	Participation bias	Selection of controls	Exposure				Outcome				Covariates		Differential loss to follow up	Exclusion of individuals with missing data			
				Differential			Non-differential	Differential			Non-differential	Differential	Non-differential					
				Recall bias	Observer bias	Ascertainment bias		Recall bias	Observer bias	Ascertainment bias								
Lin, 2010	Low: adjusted for age using 10/20 years bands, and adjusted for other potential confounders.	Low: automated participation, comparison cohort selected from a representative subsample of entire database.	N/A	Low: Exposure ascertained from pre-existing medical records - doesn't rely on patient recall in context of stroke	Low: exposure status defined before occurrence of stroke	Low: exposure diagnosis pre-dated stroke	Low: Medical diagnosis of HZ in medical records. Not clear how person time for those in comparison cohort who develop HZ in 1-year follow-up is handled. Possibly some misclassification of exposed person time.	Low: stroke identified from medical records - doesn't rely on patient recall	Low: clinical diagnosis of stroke unlikely to be severely affected by recent HZ reactivation.	Low: exposure status (HZ reactivation) unlikely to affect primary care attendance with stroke	Unclear: stroke definition not reported, however we assume its from ICD-10 codes.	Low: Capture unlikely to differ by HZ exposure status.	Low: ascertained from medical records. No information on lifestyle factors (eg smoking), however unlikely to be a significant confounder.	Low: automated follow-up	Unclear: missing data not described	Low: exposure defined prior to development of stroke.	Applicable to immunocompetent adults of Chinese ethnicity	Low: Large study with narrow confidence intervals
Liao, 2017	Moderate: categorisation of age not described. Range of confounders considered	Low: automated participation, comparison cohort selected from a representative subsample of entire database.	N/A	Low: Exposure ascertained from pre-existing medical records - doesn't rely on patient recall in context of stroke	Low: exposure status defined before occurrence of stroke	Low: exposure diagnosis pre-dated stroke	Low: Medical diagnosis of HZ in medical records. Not clear how person time for those in comparison cohort who develop HZ in 1-year follow-up is handled. Possibly some misclassification of exposed person time.	Low: stroke identified from medical records - doesn't rely on patient recall	Low: clinical diagnosis of stroke unlikely to be severely affected by recent HZ reactivation.	Low: exposure status (HZ reactivation) unlikely to affect primary care attendance with stroke	Low: Medical diagnosis of stroke.	Low: Capture unlikely to differ by HZ exposure status.	Low: ascertained from medical records.	Low: automated follow-up	Unclear: missing data not described	Low: exposure defined prior to development of stroke.	Applicable to patients with rheumatoid arthritis	Low: Large study with narrow confidence intervals
Minassian, 2015	Low: tightly controlled for age and time invariant confounders	Low: automated participation	N/A	Low: Exposure ascertained from pre-existing medical records - doesn't rely on patient recall in context of stroke	Low: exposure status defined before occurrence of stroke	Low: exposure diagnosis pre-dated stroke	Low: Medical diagnosis of HZ in medical records.	Low: stroke identified from medical records - doesn't rely on patient recall	Low: clinical diagnosis of stroke unlikely to be severely affected by recent HZ reactivation.	Low: exposure status (HZ reactivation) unlikely to affect primary care attendance with stroke	Low: Medical diagnosis of stroke with thorough exclusions to avoid misclassification.	Low: Capture unlikely to differ across risk periods	Low: adjusted for age only	Low: automated follow-up	Low: no missing data.	Low: exposure defined prior to development of stroke.	Applicable to older adults	Low: Large study with narrow confidence intervals
Patterson, 2018	High: not clear how close exposed and unexposed matched on age - not clear if age additionally adjusted for in analysis.	Low: automated participation	N/A	Low: Exposure ascertained from pre-existing medical records - doesn't rely on patient recall in context of stroke	Low: exposure status defined before occurrence of stroke	Low: exposure diagnosis pre-dated stroke	Low: Medical diagnosis of HZ in medical records.	Low: stroke identified from medical records - doesn't rely on patient recall	Low: clinical diagnosis of stroke unlikely to be severely affected by recent HZ reactivation.	Low: exposure status (HZ reactivation) unlikely to affect primary care attendance with stroke	Low: Medical diagnosis of stroke. Stroke type (ischaemic/haemorrhagic) available 93% of sample.	Low: Adjusted for age and vascular risk factors. Capture unlikely to differ across risk periods	Low: ascertained from inpatients and outpatient records.	Low: automated follow-up	Low: no missing data.	Low: exposure defined prior to development of stroke.	Applicable to adults	Low: Large study with narrow confidence intervals
Schink et al, 2016	Low: Adjusted for age using 5-year bands. SCCS design means time-invariant confounding implicitly controlled for.	Low: automated participation	N/A	Low: Exposure ascertained from pre-existing medical records - doesn't rely on patient recall in context of stroke	Low: exposure status defined before occurrence of stroke	Low: exposure diagnosis pre-dated stroke	Low: Medical diagnosis of HZ in medical records.	Low: stroke identified from medical records - doesn't rely on patient recall	Low: clinical diagnosis of stroke unlikely to be severely affected by recent HZ reactivation.	Low: exposure status (HZ reactivation) unlikely to affect primary care attendance with stroke	Low: Medical diagnosis of stroke. Stroke type (ischaemic/haemorrhagic) available 93% of sample.	Low: Adjusted for age and vascular risk factors. Capture unlikely to differ across risk periods	Low: ascertained from inpatients and outpatient records.	Low: automated follow-up	Low: no missing data.	Low: exposure defined prior to development of stroke.	Applicable to HZ patients treated with antivirals or visiting hospital for their HZ; potentially capturing more severe HZ patients.	Low: Large study with narrow confidence intervals
Sreenivasan, 2013	Moderate: categorisation of age not described. Range of confounders considered	Low: all Danish adults used as the source population. Also allowed exposed patients to contribute person time to the unexposed group, prior to HZ diagnosis.	N/A	Low: Exposure ascertained from pre-existing medical records - doesn't rely on patient recall in context of stroke	Unclear: Exposure ascertained through medical records, although not clear if observer aware of outcome status.	Low: exposure diagnosis pre-dated stroke	High: relied on acyclovir treatment as a proxy for HZ - may have resulted in misclassification with herpes simplex plus exposed patients misclassified as unexposed.	Low: stroke identified from medical records - doesn't rely on patient recall	Low: clinical diagnosis of stroke unlikely to be severely affected by recent HZ reactivation.	Low: explored whether HZ increased risk of fractures; no strong evidence of association, suggesting minimal risk of ascertainment bias.	Low: Medical diagnosis of stroke.	Low: capture unlikely to differ between exposed and unexposed	Low: ascertained from medical records. No information on lifestyle factors (eg smoking), however unlikely to be a significant confounder.	Low: automated follow-up	Unclear: missing data not described	Low: exposure defined prior to development of stroke.	Applicable to treated HZ patients (the results may not apply to untreated HZ patients).	Low: Large study with narrow confidence intervals
Sundström, 2015	High: Only adjusted for age and sex - categorisation of age not described.	Unclear: All incident cases of zoster included, however comparison population not well described.	N/A	Low: Exposure ascertained from pre-existing medical records - doesn't rely on patient recall in context of stroke	Unclear: Exposure ascertained through medical records, although not clear if observer aware of outcome status.	Low: exposure diagnosis pre-dated stroke	Low: Medical diagnosis of HZ in medical records - PPV of diagnosis 86%.	Low: stroke identified from medical records - doesn't rely on patient recall	Low: clinical diagnosis of stroke unlikely to be severely affected by recent HZ reactivation.	Low: exposure status unlikely to be associated with health-seeking behaviour and affect stroke ascertainment	Low: Medical diagnosis of stroke.	Low: capture unlikely to differ between exposed and unexposed	Low: adjusted for age and sex only	Low: automated follow-up	Unclear: missing data not described	Low: exposure defined prior to development of stroke.	Applicable to patients of any age	Low: Large study with narrow confidence intervals
Tseng, 2011	Unclear: not clear how close exposed and unexposed matched on age - not clear if age additionally adjusted for in analysis.	Low: automated participation, comparison cohort selected from a random sample of unexposed patients	N/A	Low: Exposure ascertained from pre-existing medical records - doesn't rely on patient recall in context of stroke	Low: exposure status defined before occurrence of stroke	Low: exposure diagnosis pre-dated stroke	Low: Medical diagnosis of HZ in medical records.	Low: stroke identified from medical records - doesn't rely on patient recall	Low: clinical diagnosis of stroke unlikely to be severely affected by recent HZ reactivation.	Low: exposure status unlikely to be associated with health-seeking behaviour and affect stroke ascertainment	Unclear: potential misclassification unclear due to insufficient information about data source	Unclear: ascertainment of covariates not described	Unclear: ascertainment of covariates not described	Low: automated follow-up	Unclear: missing data not described	Low: exposure defined prior to development of stroke.	Applicable to older individuals	Moderate: Modestly sized study, limiting conclusions

	Confounding	Selection of participants		Misclassification of variables										Bias due to missing data		Reverse causation	Generalisability	Study Power
	Age and other confounders	Participation bias	Selection of controls	Exposure				Outcome				Covariates		Differential loss to follow up	Exclusion of individuals with missing data			
				Differential			Non-differential	Differential			Non-differential	Differential	Non-differential					
				Recall bias	Observer bias	Ascertainment bias		Recall bias	Observer bias	Ascertainment bias								
Yawn, 2016	Low: age used in matching exposed to unexposed and included in analyses. Other confounders also included.	Low: automated participation, comparison cohort selected from a random sample of unexposed patients	N/A	Low: Exposure ascertained from pre-existing medical records - doesn't rely on patient recall in context of stroke	Low: exposure status defined before occurrence of stroke	Low: exposure diagnosis pre-dated stroke	Low: Medical diagnosis of HZ in medical records confirmed by medical record review	Low: stroke identified from medical records - doesn't rely on patient recall	Low: clinical diagnosis of stroke unlikely to be severely affected by recent HZ reactivation.	Low: exposure status unlikely to be associated with health-seeking behaviour and affect stroke ascertainment	Low: Medical diagnosis of stroke.	Low: Capture unlikely to differ by HZ exposure status.	Low: required 2 codes to be present for each covariate, however, covariates not time-updated.	Low: automated follow-up - Loss to follow up, primarily due to death in these elderly patients was almost 45% by year 5 but was less than 1% at 3 months	No missing data	Low: exposure defined prior to development of stroke.	Applicable to older individuals	Low: Large study with narrow confidence intervals
Cytomegalovirus infection																		
Coles, 2003	Moderate: categorisation of age not described. However did adjust for age and various confounders	Low: Characteristics of the random sub-cohort similar to the entire cohort, indicating that the subjects with suitable sera available were representative of the entire cohort.	Low: controls selected from the same survey cohort from which the cases arose.	Low: Exposure ascertained through laboratory assays - doesn't rely on patient recall.	Unclear: Exposure ascertained through laboratory assays, although not clear if observer aware of outcome status.	Low: Exposure ascertained through laboratory assays for all cohort.	Low: Exposure ascertained through laboratory assays for all cohort.	Low: stroke identified from medical records - doesn't rely on patient recall	Low: stroke identified from medical records; unlikely to be severely affected by exposure	Low: exposure status unlikely to affect primary care attendance with stroke	Low: Medical diagnosis of stroke.	Unclear: ascertainment of covariates not described in detail.	Unclear: ascertainment of covariates not described in detail.	Low: automated follow-up	Unclear: missing data not described	Low: exposure defined prior to ascertainment of stroke.	Applicable to older individuals with no history of cardiovascular disease.	High: This study had about 50% power to detect a relative risk of 1.5 for stroke.
Fagerberg, 1999	High: no adjustment for age. Some other confounders considered.	Moderate: random sample of an existing cohort: of the 164/508 (32%) randomly selected from the original cohort, 152 (93%) had blood samples available. Although no comparison of those from substudy with/without blood samples, very few excluded.	N/A	Low: Exposure ascertained through laboratory assays - doesn't rely on patient recall.	Unclear: Exposure ascertained through laboratory assays, although not clear if observer aware of outcome status.	Low: Exposure ascertained through laboratory assays for all cohort.	Unclear: definition of high/low antibody titres to CMV not provided	Low: stroke identified from diagnostic imaging - doesn't rely on patient recall.	Low: all events coded by physicians blinded to patient identity	Low: exposure status unlikely to affect primary care attendance with stroke	Low: Medical diagnosis of stroke confirmed by 2 physicians	Low: Capture unlikely to differ by CMV exposure status.	Low: good ascertainment of covariates	Low: No patient was lost to follow-up	Unclear: missing data not described	Low: exposure defined prior to development of stroke.	Applicable to men over 50 years old	High: small study, power calculation not described, wide confidence intervals.
González-Quijad, 2015	Moderate: Age adjusted using a binary variable, however a range of confounders were included in the multivariate model	Moderate: cases selected randomly using a computer program - no information given on numbers excluded (eg patients refusing to participate).	High: no description of how controls were selected.	Low: Exposure ascertained through laboratory assays - doesn't rely on patient recall.	Low: exposure classified by laboratory staff unaware of any patient details	Low: Exposure ascertained through laboratory assays for all cohort.	Low: Exposure ascertained through laboratory assays for all cohort.	Low: stroke classified by physicians blinded to serological results - doesn't rely on patient recall.	Low: stroke classified by physicians blinded to serological results.	Low: exposure status unlikely to affect secondary care attendance with stroke	Low: stroke diagnoses confirmed by medical professionals and verified with diagnostic images	Low: Capture unlikely to differ by CMV exposure status.	Low: good ascertainment of covariates	Low: case-control study, therefore no follow-up.	Unclear: missing data not described	High: exposure defined after the stroke defined as the study outcome.	Applicable to older individuals.	Moderate: Study powered for a different exposure (Coxiella Burnetii), however despite the small study, the confidence intervals were relatively narrow.
Huang, 2012	Low: Matched on age within 3 years, adjusted for various risk factors.	Moderate: random sample of an existing cohort, no comparison of those included versus excluded in this sub study.	Low: controls selected from same cohort as cases and matched to cases on geographical region.	Low: Exposure ascertained through laboratory assays - doesn't rely on patient recall.	Unclear: Exposure ascertained through laboratory assays, although not clear if observer aware of case/control status.	Low: Exposure ascertained through laboratory assays for all cohort.	Low: Exposure ascertained through laboratory assays for all cohort.	Low: stroke identified from diagnostic imaging - doesn't rely on patient recall.	Low: clinical diagnosis of stroke occurred in hospital, prior to exposure status being known.	Low: exposure status unlikely to affect primary care attendance with stroke	Low: Medical diagnosis of stroke confirmed through diagnostic images	Unclear: ascertainment of covariates not described in detail.	Unclear: ascertainment of covariates not described in detail.	Low: case-control study, therefore no follow-up.	Unclear: missing data not described	High: exposure defined after the stroke defined as the study outcome.	Applicable to people of Chinese Han ethnicity	Moderate: Small study, however confidence intervals were narrow.
Kenina, 2010	High: no adjustment for any potential confounders	Unclear: not clear how sample were recruited	High: no description of how controls were selected.	Low: Exposure ascertained through laboratory assays - doesn't rely on patient recall.	Unclear: Exposure ascertained through laboratory assays, although not clear if observer aware of case/control status.	Low: Exposure ascertained through laboratory assays for all cohort.	Low: Exposure ascertained through laboratory assays for all cohort.	Low: stroke patients recruited from a Neurology clinic - doesn't rely on patient recall.	Low: clinical diagnosis of stroke occurred in hospital, prior to exposure status being known.	Low: exposure status unlikely to affect primary care attendance with stroke	Unclear: no discussion of how stroke was confirmed	No covariates	No covariates	Low: case-control study, therefore no follow-up.	Unclear: missing data not described	High: exposure defined after the stroke defined as the study outcome.	Study population of interest unclear	High: small study, power calculation not described, wide confidence intervals.
Oliveras, 2003	High: no adjustment for any potential confounders	Unclear: not clear how sample were recruited	N/A	Unclear: not clear how exposure ascertained.	Unclear: not clear how exposure ascertained.	Unclear: not clear how exposure ascertained.	High: very low proportion of cohort were classified as having CMV infection.	Low: stroke defined from symptoms and imaging data - didn't rely on patient recall	Unclear: stroke defined from symptoms and imaging data - unclear if observers aware of CMV status	Low: exposure status unlikely to affect primary care attendance with stroke	Low: stroke defined from symptoms and imaging data	No covariates	No covariates	Low: 27 patients (6.3%) lost to follow-up	Unclear: missing data not described	Low: exposure defined prior to ascertainment of stroke.	Applicable to renal transplant recipients	Low: very few patients with CMV infection
Shen, 2011	High: no adjustment for any potential confounders	High: non-random sample, no description of numbers not participating, no comparison of those included versus enrolled.	Low: controls selected from the hospital where the case arose.	Low: Exposure ascertained through laboratory assays - doesn't rely on patient recall.	Unclear: Exposure ascertained through laboratory assays, although not clear if observer aware of outcome status.	Low: Exposure ascertained through laboratory assays for all cohort.	Low: Exposure ascertained through laboratory assays for all cohort.	Low: stroke defined before ascertainment of exposure status - doesn't rely on patient recall.	Unclear: unclear if observers aware of exposure status	Low: exposure status unlikely to affect primary care attendance with stroke	Low: stroke defined from symptoms and imaging data	No covariates	No covariates	Low: case-control study, therefore no follow-up.	Unclear: missing data not described	High: exposure defined after the stroke defined as the study outcome.	Applicable to older individuals	High: small study, power calculation not described, wide confidence intervals.

	Confounding	Selection of participants		Misclassification of variables										Bias due to missing data		Reverse causation	Generalisability	Study Power
				Exposure				Outcome				Covariates						
				Differential			Non-differential	Differential			Non-differential	Differential	Non-differential					
	Age and other confounders	Participation bias	Selection of controls	Recall bias	Observer bias	Ascertainment bias		Recall bias	Observer bias	Ascertainment bias				Differential loss to follow up	Exclusion of individuals with missing data			
Smieja, 2003	Moderate: categorisation of age not described. However did adjust for age and various confounders	Low: Only 3168/9541 (33%) participants had sufficient blood available for analysis, however authors comment this is a random subsample and sample is similar to overall HOPE study.	N/A	Low: Exposure ascertained through laboratory assays - doesn't rely on patient recall.	Unclear: Exposure ascertained through laboratory assays, although not clear if observer aware of outcome status.	Low: Exposure ascertained through laboratory assays for all cohort.	Low: Exposure ascertained through laboratory assays - only 0.3% of participants had indeterminate values for CMV status.	Low: stroke defined before ascertainment of CMV status - doesn't rely on patient recall.	Unclear: unclear if observers aware of CMV status	Low: exposure status unlikely to affect primary care attendance with stroke	Unclear: very little discussion of how stroke was confirmed	Unclear: ascertainment of covariates not described in detail.	Unclear: ascertainment of covariates not described in detail.	Unclear: loss to follow-up not described.	Unclear: Missing data not described, except for CMV status where 15/3153 (0.5%) patients had indeterminate values.	Low: exposure defined prior to ascertainment of stroke.	Applicable to older individuals with a history of cardiovascular disease	High: Large study, however confidence intervals were wide. No power calculation described and stroke alone was a secondary outcome.
Tarnacka, 2002	High: no adjustment for any potential confounders	High: consecutive sampling and no information on eligible patients not included in the analyses (eg refusing to participate)	High: no description of how controls were selected.	Low: Exposure ascertained through laboratory assays - doesn't rely on patient recall.	Unclear: Exposure ascertained through laboratory assays, although not clear if observer aware of outcome status.	Low: Exposure ascertained through laboratory assays for all cohort.	Low: Exposure ascertained through laboratory assays for all cohort.	Low: stroke defined before ascertainment of CMV status - doesn't rely on patient recall.	Unclear: unclear if observers aware of CMV status	Low: exposure status (CMV infection) unlikely to affect primary care attendance with stroke	Low: stroke defined from symptoms and imaging data	No covariates	No covariates	Low: case-control study, therefore no follow-up.	Unclear: missing data not described	High: exposure defined after the stroke defined as the study outcome.	Study population of interest unclear	High: small study, power calculation not described, wide confidence intervals.
Yi, 2008	High: no adjustment for any potential confounders (and matching factors not accounted for in the analysis).	Unclear: not clear how sample were recruited	High: no description of how controls were selected. Excluded controls with IgG antibodies to CMV - not clear if same exclusion criteria applied to cases.	Low: Exposure ascertained through laboratory assays - doesn't rely on patient recall.	Unclear: Exposure ascertained through laboratory assays, although not clear if observer aware of outcome status.	Low: Exposure ascertained through laboratory assays for all cohort.	Low: Exposure ascertained through laboratory assays for all cohort.	Low: stroke defined before ascertainment of CMV status - doesn't rely on patient recall.	Unclear: unclear if observers aware of CMV status	Low: exposure status (CMV infection) unlikely to affect primary care attendance with stroke	Low: stroke defined from symptoms and imaging data	No covariates	No covariates	Low: case-control study, therefore no follow-up.	Low: no missing data.	High: exposure defined after the stroke defined as the study outcome.	Applicable to older individuals	High: small study, power calculation not described, wide confidence intervals.
Zheng, 2016	Low: good adjustment for age and other confounders	Low: randomly selected cohort of patients, using stratified cluster, multistage sampling method	Low: controls selected from the same survey cohort from which the cases arose.	Low: Exposure ascertained through laboratory assays - doesn't rely on patient recall.	Low: exposure defined by laboratory staff blinded to case-control status	Low: exposure diagnosis pre-dated stroke	Low: Exposure ascertained through laboratory assays for all cohort.	Low: stroke identified from medical records - doesn't rely on patient recall.	Low: stroke defined by investigators blinded to exposure status	Low: exposure status unlikely to affect primary care attendance with stroke	Low: Medical diagnosis of stroke.	Low: covariates ascertained similarly for exposed and unexposed	Low: ascertainment of covariates by local trained doctors using home visits	Low: 634/6412 (9.9%) of original survey lost to follow-up	Unclear: missing data not described	Low: exposure defined prior to development of stroke.	Applicable to Chinese people living in rural areas with hypertension	Moderate: small study, power calculation not described.
Ziemann, 2016	High: no adjustment for any potential confounders	Moderate: 16.6% of eligible participants excluded due to inadequate sample. Not clear how these participants differed from those included.	N/A	Low: Exposure ascertained through laboratory assays - doesn't rely on patient recall.	Unclear: exposure defined by laboratory staff - unclear if blinded to outcome status	Low: exposure diagnosis pre-dated stroke	Low: Exposure ascertained through laboratory assays for all cohort.	Low: stroke identified from medical records - doesn't rely on patient recall.	Unclear: unclear if stroke defined by investigators blinded to exposure status	Low: exposure status unlikely to affect primary care attendance with stroke	Moderate: Medical diagnosis of stroke, though unclear if capture in cardiac surgery database is complete.	No covariates	No covariates	Low: 98.3% of participants followed up.	Low: no missing data.	Low: exposure defined prior to development of stroke.	Applicable to older patients who have undergone cardiac surgery	High: small study, wide confidence intervals, power calculation not described.
Cytomegalovirus reactivation																		
Yen, 2016	Low: good adjustment for age and other confounders	Low: automated participation	N/A	Low: Exposure ascertained from pre-existing medical records; doesn't rely on patient recall.	Low: used pre-specified codes before occurrence of stroke	Low: exposure diagnosis pre-dated stroke	Low: Medical diagnoses in medical records.	Low: stroke identified from medical records - doesn't rely on patient recall	Low: clinical diagnosis of stroke in hospital unlikely to be severely affected by previous CMV	Low: exposure status unlikely to affect primary care attendance with stroke	Low: defined from medical records which high sensitivity and specificity	Low: covariates ascertained similarly for exposed and unexposed	Low: ascertained from inpatients and outpatient records using ICD-9 codes.	Low: automated follow-up	Low: no missing data.	Low: exposure defined prior to development of stroke.	Applicable to patients with HIV infection, and largely a male population.	Moderate: Moderate-sized study, wide confidence intervals, power calculation not described.
HHV6																		
Fullerton, 2017	High: no adjustment for any potential confounders (and matching factors not accounted for in the analysis).	Unclear: not clear how sample were recruited	Moderate: controls selected from the population giving rise to the cases (same hospital), however not clear whether controls were recruited randomly	Low: Exposure ascertained through laboratory assays - doesn't rely on patient recall.	Unclear: exposure defined by laboratory staff - unclear if blinded to outcome status	Low: Exposure ascertained through laboratory assays for all cohort.	Low: Exposure ascertained through laboratory assays for all cohort.	Low: stroke status defined prior to exposure status known, doesn't rely on patient recall.	Low: stroke status defined prior to exposure status known	Low: ascertainment of stroke unlikely to be influenced by herpesvirus status.	Unclear: stroke definition not described	No covariates	No covariates	Low: case-control study, therefore no follow-up.	Unclear: missing data not described	High: exposure defined after the stroke defined as the study outcome.	Applicable to children with stroke	High: small study, power calculation not described.
VZV infection, serologically defined																		
Asikiet al, 2015	Moderate: matched cases and controls on sex and age, however age bands for matching not described. No other confounders considered.	High: non-random sample, no description of numbers not participating, no comparison of those included versus enrolled.	Low: controls selected from the village where the case arose.	Low: Exposure ascertained through laboratory assays - doesn't rely on patient recall.	Low: Exposure ascertained through laboratory assays by individuals blinded to case status.	Low: Exposure ascertained through laboratory assays for all cohort.	Low: Exposure ascertained through laboratory assays for all cohort.	Low: stroke status defined prior to exposure status known, doesn't rely on patient recall.	Low: stroke status defined prior to exposure status known	Low: ascertainment of stroke unlikely to be influenced by herpesvirus status.	Moderate: stroke defined from clinical symptoms and verbal autopsy only	Low: Capture unlikely to differ by case/control status.	Low: Matched on age and sex only.	Low: case-control study, therefore no follow-up.	Low: no missing data.	Low: exposure defined prior to development of stroke.	Applicable to older, rural African communities.	High: small study, power calculation not described.
VZV infection, clinically defined																		

	Confounding	Selection of participants		Misclassification of variables										Bias due to missing data		Reverse causation	Generalisability	Study Power
	Age and other confounders	Participation bias	Selection of controls	Exposure				Outcome				Covariates		Differential loss to follow up	Exclusion of individuals with missing data			
				Differential			Non-differential	Differential			Non-differential	Differential	Non-differential					
				Recall bias	Observer bias	Ascertainment bias		Recall bias	Observer bias	Ascertainment bias								
Askalan, 2001	High: no adjustment for confounders	High: non-random sample, no description of numbers not participating, no comparison of those included versus enrolled.	N/A	High: exposure status defined by parent recall of exposure in context of recent stroke	Low: exposure status defined by parents, rather than observer	Low: exposure ascertained for all cohort	High: relied on memory recall of previous 12 months	Unclear: unclear when recurrent stroke status defined relative to exposure status	Unclear: unclear when recurrent stroke status defined relative to exposure status	Low: exposure status (HZ reactivation) unlikely to affect ascertainment of recurrent stroke	Low: active data collection of recurrent stroke via various data sources.	No covariates	No covariates	Low: little follow-up required	Low: no missing data.	Low: captured exposure status prior to recurrent stroke.	Applicable to children with stroke	High: small study, power calculation not described.
Sébire, 1999	Moderate: matched cases and controls on sex, age and site, but not accounted for in the analysis. No other confounders considered.	Low: all children referred for stroke treatment during study period	High: no description of how controls were selected.	Low: Exposure ascertained from pre-existing medical records - doesn't rely on patient recall in context of stroke	Unclear: Exposure ascertained through medical records, although not clear if observer aware of outcome status.	Low: exposure diagnosis pre-dated stroke	High: varicella may not have required medical examination, therefore may not appear in medical records.	Low: stroke identified from diagnostic imaging - doesn't rely on patient recall.	Low: stroke defined from symptoms and imaging data - unclear if observers aware of varicella status	Low: exposure status unlikely to affect secondary care attendance with stroke	Low: Medical diagnosis of stroke confirmed through diagnostic images	No covariates	No covariates	Low: case-control study, therefore no follow-up.	Unclear: missing data not described	Low: exposure defined prior to development of stroke.	Applicable to children	High: small study, power calculation not described, wide confidence intervals.
Thomas, 2014	Low: good adjustment for age (using 5-year age bands) and other confounders	Low: automated participation	N/A	Low: Exposure ascertained from pre-existing medical records - doesn't rely on patient recall in context of stroke	Low: exposure status defined before occurrence of stroke	Low: exposure diagnosis pre-dated stroke	Low: although varicella may not always be recorded in primary care, by using a SCCS, the authors avoid non-differential misclassification of the exposure.	Low: stroke identified from medical records based on hospital visits - doesn't rely on patient recall	Low: clinical diagnosis of stroke unlikely to be severely affected by recent varicella.	Low: varicella may be associated with health-seeking behaviour, and associated with greater chance of stroke being recorded in primary care.	Low: Medical diagnosis of stroke. May be some misclassification with haemorrhagic stroke as most strokes were classified as non-specific.	Low: Capture unlikely to differ by exposure status.	Age was the only covariate and would be well captured in primary care	Low: automated follow-up	No missing data	Low: exposure defined prior to development of stroke.	Applicable to patients who would present with varicella to their general practitioner	Moderate: Modestly sized study, limiting conclusion among adult population
Vaccination against herpesviruses (e.g. zostavax vaccine)																		
Baxter, 2012	Low: no adjustment for confounders, but design accounts for within person confounding	Low: automated participation	N/A	Low: Exposure ascertained from pre-existing medical records - doesn't rely on patient recall in context of stroke	Low: used pre-specified codes before occurrence of stroke	Low: exposure diagnosis pre-dated stroke	Low: designated system to record vaccinations	Low: stroke identified from medical records - doesn't rely on patient recall	Low: clinical diagnosis of stroke unlikely to be severely affected by recent HZ vaccination.	Low: exposure status (HZ vaccination) unlikely to affect primary care attendance with stroke	Low: Medical diagnosis of stroke.	No covariates	No covariates	Low: 1.6% of vaccinated patients excluded due to lack of adequate follow-up.	Low: no missing data.	Low: no missing data.	Applicable to patients aged ≥ 60 years receiving HZ vaccination.	Moderate: large number of vaccinated patients, but very few stroke events.
Kovac, 2018	Low: randomised design accounts for confounders	Low: randomised participation	N/A	Low: exposure randomised prior to stroke being recorded	Low: exposure randomised (blinded to observers) prior to stroke being recorded	Low: exposure randomised prior to stroke being recorded	Low: exposure randomised	Low: stroke identified by specialists - doesn't rely on patient recall	Low: observers blinded to exposure status	Low: exposure status (HZ vaccination) unlikely to affect primary care attendance with stroke	Low: Medical diagnosis of stroke.	Low: randomised design	Low: randomised design	Unclear: follow-up not described	Low: no missing data.	Low: no missing data.	Applicable to older individuals	Moderate: large number of vaccinated patients, but very few stroke events.
Tseng, 2012	Low: no adjustment for confounders, but design accounts for within person confounding	Low: automated participation	N/A	Low: Exposure ascertained from pre-existing medical records - doesn't rely on patient recall in context of stroke	Low: exposure status defined before occurrence of stroke	Low: exposure diagnosis pre-dated stroke	Low: although HZ vaccination may not always be recorded, by using a SCCS, the authors avoid non-differential misclassification of the exposure.	Low: stroke identified from medical records - doesn't rely on patient recall	Low: clinical diagnosis of stroke unlikely to be severely affected by recent HZ vaccination	Low: exposure status (HZ vaccination) unlikely to affect primary care attendance with stroke	Low: Medical diagnosis of stroke.	No covariates	No covariates	Low: automated follow-up	No missing data	Low: exposure defined prior to development of stroke.	Applicable to older individuals	Moderate: Modestly sized study, limiting conclusion among adult population
Donahue, 2009	Low: good adjustment for age and other confounders	Low: automated participation	N/A	Low: Exposure ascertained from pre-existing medical records - doesn't rely on patient recall in context of stroke	Low: used pre-specified codes before occurrence of stroke	Low: exposure diagnosis pre-dated stroke	Low: designated system to record vaccinations	Low: stroke identified from medical records - doesn't rely on patient recall	Low: clinical diagnosis of stroke in hospital unlikely to be severely affected by recent varicella vaccination.	Low: exposure status (varicella vaccination) unlikely to affect primary care attendance with stroke	Low: Medical diagnoses of stroke may be misclassified as other stroke-like conditions, eg migraine, as rare as difficult to diagnose in children.	Low: Adjusted for age and vascular risk factors. Capture unlikely to differ by vaccinated/unvaccinated or across risk periods among vaccinated.	Low: ascertained from inpatients and outpatient records using ICD-9 codes.	Low: automated follow-up	Unclear: missing data not described	Low: exposure defined prior to development of stroke.	Applicable to immunocompetent children aged >11 months up to 18 years.	High: authors estimated the power to be 80% to detect a relative risk of 1.7 for stroke during a 9-month post-vaccination period. Relative risk may be much smaller than this.
MacDonald, 2018	Unclear: not clear if age adjusted for in analysis.	Low: automated participation	N/A	Low: Exposure ascertained from pre-existing medical records - doesn't rely on patient recall in context of stroke	Low: used pre-specified codes before occurrence of stroke	Low: vaccination pre-dated stroke	Low: designated system to record vaccinations	Low: stroke identified from medical records - doesn't rely on patient recall	Low: clinical diagnosis of stroke in hospital unlikely to be severely affected by recent varicella vaccination.	Low: exposure status (varicella vaccination) unlikely to affect primary care attendance with stroke	Low: Medical diagnoses of stroke may be misclassified as other stroke-like conditions, eg migraine, as rare as difficult to diagnose in children.	Low: Adjusted for age and vascular risk factors. Capture unlikely to differ by vaccinated/unvaccinated or across risk periods among vaccinated.	Low: ascertained from inpatients and outpatient records using ICD-9 codes.	Low: automated follow-up	Unclear: missing data not described	Low: exposure defined prior to development of stroke.	Applicable to aged >11 months up to 23 months.	Moderate: large number of vaccinated patients, but very few stroke events.
Multiple herpes viruses																		

	Confounding	Selection of participants		Misclassification of variables										Bias due to missing data		Reverse causation	Generalisability	Study Power
	Age and other confounders	Participation bias	Selection of controls	Exposure				Outcome				Covariates		Differential loss to follow up	Exclusion of individuals with missing data			
				Differential			Non-differential	Differential			Non-differential	Differential	Non-differential					
				Recall bias	Observer bias	Ascertainment bias		Recall bias	Observer bias	Ascertainment bias								
Al-Ghamdi, 2012	High: although cases were matched on age and gender, the matching was not accounted for in the analysis. No other variables investigated as confounders	Unclear: not clear how sample were recruited	High: no description of how controls were selected.	Low: Exposures ascertained through laboratory assays - doesn't rely on patient recall.	Unclear: Exposure ascertained through laboratory assays, although not clear if observer aware of outcome status.	Low: Exposure ascertained through laboratory assays for all cohort.	Low: Exposure ascertained through laboratory assays for all cohort.	Unclear: stroke definition not described	Unclear: stroke definition not described	Unclear: stroke definition not described	Unclear: stroke definition not described	No covariates	No covariates	Low: case-control study, therefore no follow-up.	Unclear: missing data not described	Unclear: stroke definition not described	Applicable to patients attending a hospital in Saudi Arabia	High: small study, wide confidence intervals, power calculation not described.
Elkind, 2010	Low: good adjustment for age and other confounders	Low: Characteristics of 1625 participants analysed similar to entire cohort; subjects with suitable sera were representative of entire cohort.	N/A	Low: Exposure ascertained through laboratory assays - doesn't rely on patient recall.	Low: Exposure ascertained through laboratory assays by individuals blinded to case status.	Low: Exposure ascertained through laboratory assays for all cohort.	Low: Exposure ascertained through laboratory assays for all cohort.	Low: infection with herpesvirus unlikely to affect recall of stroke symptoms	Unclear: unclear when infection status known to neurologists defining stroke status.	Low: exposure status (various herpesvirus infections) unlikely to affect primary care attendance with stroke	Moderate: based on symptom recall by patients and subsequent review by specialists.	Low: covariates ascertained similarly for exposed and unexposed	Unclear: ascertainment of covariates not described in detail.	Unclear: authors say there was minimal loss to follow-up, but no numbers are reported.	Unclear: missing data not described	Low: exposure defined prior to development of stroke.	Applicable to patients aged >39 years.	High: small study, wide confidence intervals, power calculation not described.
Elkind et al, 2016	Low: adjusted for age using categorical variable with narrow groups	Moderate: all those presenting with stroke were screened, reasons for exclusion not given, no comparison of those included versus enrolled.	Moderate: controls selected from the population giving rise to cases; but some centres did not commonly care for paediatric trauma patients thus could not enrol trauma controls; most controls from US, Canada and Chile.	Low: Exposure ascertained through laboratory assays - doesn't rely on patient recall.	Low: Exposure ascertained through laboratory assays by individuals blinded to case status.	Low: Exposure ascertained through laboratory assays for all cohort.	Moderate: Single acute blood samples had low sensitivity (suggesting high number of false negatives), there may be under ascertainment of herpesvirus infections.	Low: stroke status defined prior to herpesviruses status known	Low: stroke status defined prior to herpesviruses status known	Low: exposure status unlikely to affect primary care attendance with stroke	Low: clinical and imaging data reviewed by a medical specialist	Low: covariates ascertained similarly for cases and controls	Unclear: ascertainment of covariates not described in detail.	Low: follow-up only for 3 weeks (between enrolment and blood test for herpesviruses). 29/355 cases (8%) and 5/120 controls (4%) were excluded due to inadequate blood samples.	Low: no missing data.	High: exposure defined after the stroke defined as the study outcome.	Applicable to children, mainly from high-income settings	Moderate. Refers to a power calc that needed 120 controls (here 115 were recruited) but details not given. Numbers seem pretty small
Kis, 2007	Moderate: adjusted for a number of covariates, including age (although unclear how age was categorised).	Unclear: not clear how sample were recruited	Moderate: controls selected from the population giving rise to the cases (same hospital), however not clear whether controls were recruited randomly and controls had different age criteria.	Low: Exposure ascertained through laboratory assays - doesn't rely on patient recall.	Unclear: Exposure ascertained through laboratory assays, although not clear if observer aware of outcome status.	Low: Exposure ascertained through laboratory assays for all cohort.	Low: Exposure ascertained through laboratory assays for all cohort.	Low: stroke status defined prior to herpesviruses status known	Low: stroke status defined prior to herpesviruses status known	Low: exposure status unlikely to affect primary care attendance with stroke	Low: clinical and imaging data used to confirm stroke cases	Unclear: ascertainment of covariates not described in detail.	Unclear: ascertainment of covariates not described in detail.	Low: case-control study, therefore no follow-up.	Unclear: missing data not described	High: exposure defined after the stroke defined as the study outcome.	Applicable to patients who would attend hospital for serious illness	High: small study, wide confidence intervals, power calculation not described.
Li, 2005	High: cases were not matched to controls. No other variables investigated as confounders.	High: non-random sample, no description of numbers not participating, no comparison of those included versus enrolled.	Moderate: controls selected from the population giving rise to the cases (same hospital), however not clear whether controls were recruited randomly and controls had different mean age.	Low: Exposure ascertained through laboratory assays - doesn't rely on patient recall.	Unclear: Exposure ascertained through laboratory assays, although not clear if observer aware of outcome status.	Low: Exposure ascertained through laboratory assays for all cohort.	Low: Exposure ascertained through laboratory assays for all cohort.	Low: stroke status defined prior to herpesviruses status known	Low: stroke status defined prior to herpesviruses status known	Low: exposure status unlikely to affect primary care attendance with stroke	Low: clinical and imaging data used to confirm stroke cases	No covariates	No covariates	Low: case-control study, therefore no follow-up.	Unclear: missing data not described	High: exposure defined after the stroke defined as the study outcome.	Applicable to patients who would attend hospital for serious illness	High: small study, wide confidence intervals, power calculation not described.
Ozturk, 2013	Moderate: matched on age, no adjustment for other confounders	Unclear: not clear how sample were recruited	High: no description of how controls were selected.	Low: Exposure ascertained through laboratory assays - doesn't rely on patient recall.	Unclear: Exposure ascertained through laboratory assays, although not clear if observer aware of case/control status.	Low: Exposure ascertained through laboratory assays for all cohort.	Low: Exposure ascertained through laboratory assays for all cohort.	Low: stroke status defined prior to herpesviruses status known	Low: stroke status defined prior to herpesviruses status known	Low: exposure status unlikely to affect primary care attendance with stroke	Low: clinical and imaging data used to confirm stroke cases	No covariates	No covariates	Low: case-control study, therefore no follow-up.	Unclear: missing data not described	High: exposure defined after the stroke defined as the study outcome.	Applicable to older adults	High: small study, wide confidence intervals, power calculation not described.
Ridker, 1998	Low: good adjustment for age and other confounders	High: cohort selected from the 68% of original cohort with baseline blood data, no comparison of those with/without data.	N/A	Low: Exposure ascertained through laboratory assays - doesn't rely on patient recall.	Low: Exposure ascertained through laboratory assays by individuals blinded to case status.	Low: Exposure ascertained through laboratory assays for all cohort.	Low: Exposure ascertained through laboratory assays for all cohort.	Low: stroke identified from medical records - doesn't rely on patient recall	Low: clinical diagnosis of stroke unlikely to be severely affected by recent exposures.	Low: exposure status unlikely to affect primary care attendance with stroke	Low: defined from medical records	Unclear: ascertainment of covariates not described in detail.	Unclear: ascertainment of covariates not described in detail.	Unclear: numbers lost to follow-up not reported	Unclear: missing data not described	Low: exposure defined prior to development of stroke.	Applicable to healthy males	High: small study, wide confidence intervals, power calculation not described.

	Confounding	Selection of participants		Misclassification of variables										Bias due to missing data		Reverse causation	Generalisability	Study Power
	Age and other confounders	Participation bias	Selection of controls	Exposure				Outcome				Covariates		Differential loss to follow up	Exclusion of individuals with missing data			
				Differential			Non-differential	Differential			Non-differential	Differential	Non-differential					
				Recall bias	Observer bias	Ascertainment bias		Recall bias	Observer bias	Ascertainment bias								
Sealy-Jefferson, 2013	Low: good adjustment for age and other confounders	Low: random surveyed sample.	N/A	Low: Exposure ascertained through laboratory assays - doesn't rely on patient recall.	Unclear: Exposure ascertained through laboratory assays, although not clear if observer aware of case status.	Low: Exposure ascertained through laboratory assays for all cohort.	Low: Although pathogen data was carried forward from previous follow-up visits when missing, there was good agreement between previous and subsequent sero-status and antibody levels to each pathogen	Low: Stroke self-reported, however unlikely to be influenced by herpesvirus status (if known).	Low: self-reported stroke unlikely to be severely affected by previous herpesviruses exposures.	Low: exposure status unlikely to affect primary care attendance with stroke	High: self-reported stroke: may be misclassified.	Low: covariates ascertained similarly for exposed and unexposed	Moderate: ascertained from self-report	Low: 5.4% attrition per year of follow-up	Unclear: missing data not described	Low: exposure defined prior to development of stroke.	Applicable to older individuals of Mexican American heritage	Moderate: Moderate-sized study, wide confidence intervals, power calculation not described.
Yen, 2017	Low: good adjustment for age and other confounders	Low: automated participation	N/A	Low: Exposure ascertained through laboratory assays - doesn't rely on patient recall.	Low: used pre-specified codes before occurrence of stroke	Low: exposure diagnosis pre-dated stroke	Low: Medical diagnoses in medical records.	Low: stroke identified from medical records - doesn't rely on patient recall	Low: clinical diagnosis of stroke unlikely to be severely affected by previous herpesviruses exposures.	Low: exposure status unlikely to affect primary care attendance with stroke	Low: defined from medical records which high sensitivity and specificity	Low: covariates ascertained similarly for exposed and unexposed	Low: ascertained from inpatients and outpatient records using ICD-9 codes.	Low: automated follow-up	Low: no missing data.	Low: exposure defined prior to development of stroke.	Applicable to patients with HIV infection, and largely a male population.	Moderate: Moderate-sized study, wide confidence intervals, power calculation not described.

	Confounding	Selection of participants		Misclassification of variables										Bias due to missing data		Reverse causation	Generalisability	Study Power
	Age and other confounders	Participation bias	Selection of controls	Exposure				Outcome				Covariates		Differential loss to follow up	Exclusion of individuals with missing data			
				Differential			Non-differential	Differential			Non-differential	Differential	Non-differential					
				Recall bias	Observer bias	Ascertainment bias		Recall bias	Observer bias	Ascertainment bias								
